# Supplementary material for: The MsrAB reducing pathway of Streptococcus gordonii is needed for oxidative stress tolerance, biofilm formation, and oral colonization in mice
Source: PLoS One. 2020 Feb 21;15(2):e0229375. doi: 10.1371/journal.pone.0229375 (PMC7034828; doi:10.1371/journal.pone.0229375)
Supplement: S2 Fig — M: Prestained protein markers. (PDF) [file pone.0229375.s002.pdf]

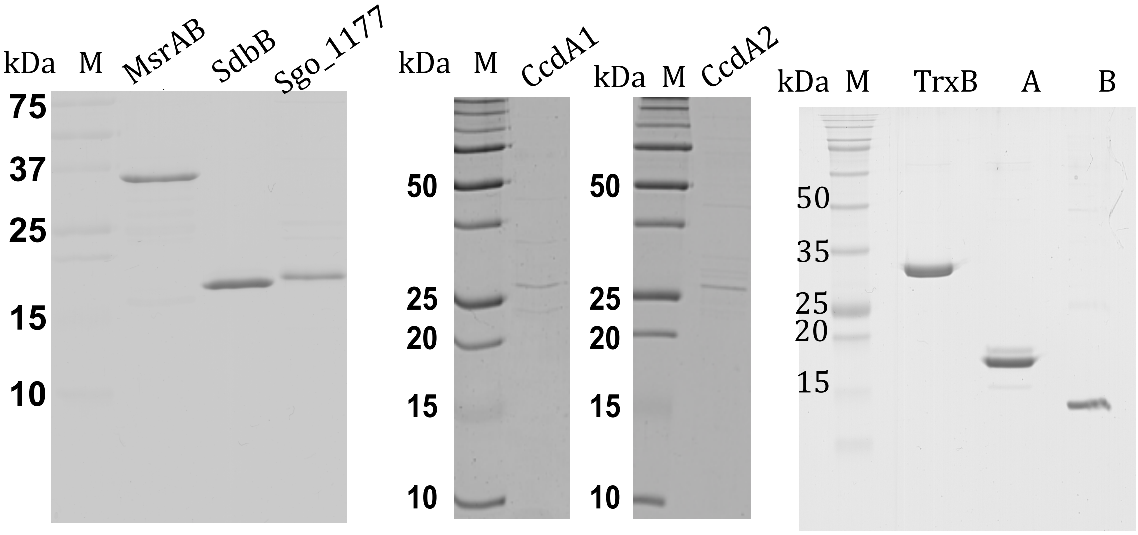

S2 Fig. SDS-PAGE of recombinant MsrAB, SdbB, Sgo_1177, CcdA1, CcdA2, TrxB, A domain of MsrAB, and B domain of MsrAB. M: Prestained protein markers.
