# Supplementary material for: The MsrAB reducing pathway of Streptococcus gordonii is needed for oxidative stress tolerance, biofilm formation, and oral colonization in mice
Source: PLoS One. 2020 Feb 21;15(2):e0229375. doi: 10.1371/journal.pone.0229375 (PMC7034828; doi:10.1371/journal.pone.0229375)
Supplement: S3 Fig — (PDF) [file pone.0229375.s003.pdf]

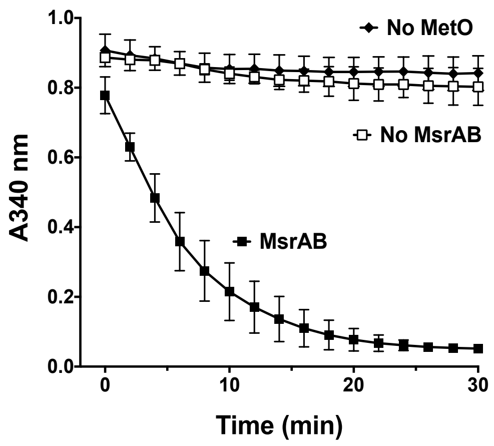

S3 Fig. Lack reduction of methionine sulfoxide when MsrAB or methionine sulfoxide (MetO) are omitted in reactions.
