# Supplementary material for: The MsrAB reducing pathway of Streptococcus gordonii is needed for oxidative stress tolerance, biofilm formation, and oral colonization in mice
Source: PLoS One. 2020 Feb 21;15(2):e0229375. doi: 10.1371/journal.pone.0229375 (PMC7034828; doi:10.1371/journal.pone.0229375)
Supplement: S4 Fig — Antibodies used were mouse sera: anti-SdbB (1/500), anti-Sgo_1177 (1/1000), anti-MsrAB (1/500), and anti-PsrA (1/2000). PrsA is an unrelated lipoprotein and served as a loading control. CBB: Coomassie-blue stained SDS-PAGE gels showing amount of proteins in samples. (PDF) [file pone.0229375.s004.pdf]

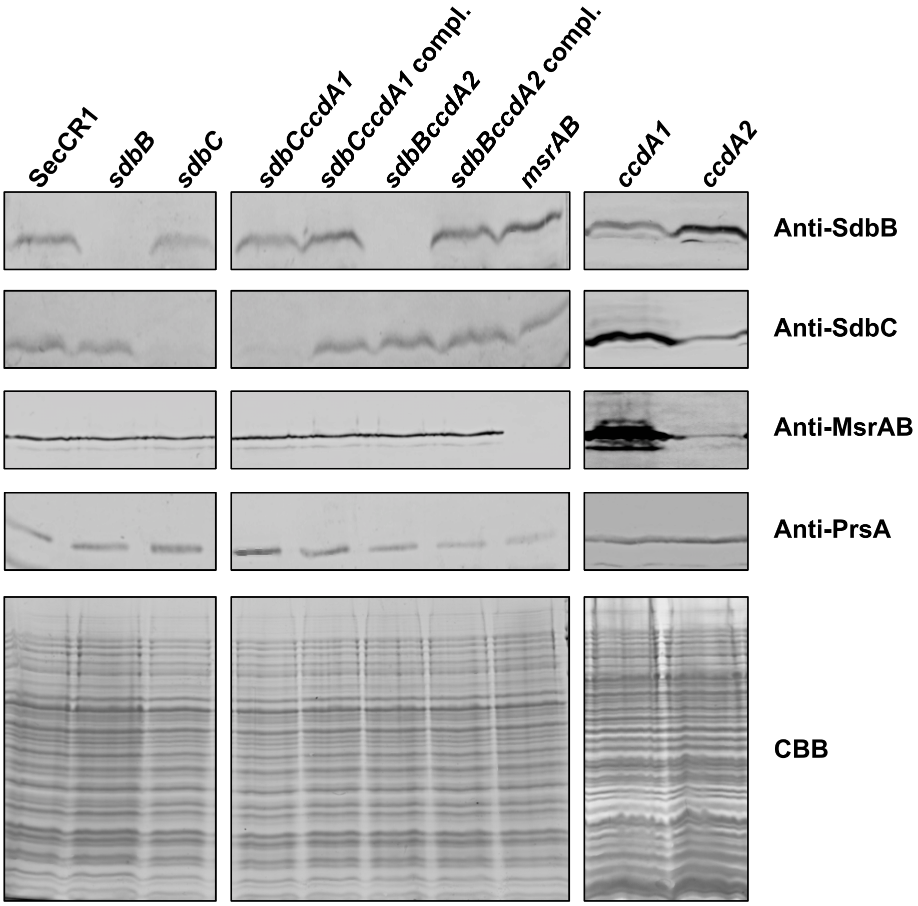

S4 Fig. Immunoblotting of *S. gordonii* mutant strains confirming the gene-knockout phenotypes. Antibodies used were mouse sera: anti-SdbB (1/500), anti-Sgo_1177 (1/1000), anti-MsrAB (1/500), and anti-PsrA (1/2000). PrsA is an unrelated lipoprotein and served as a loading control. CBB: Coomassie-blue stained SDS-PAGE gels showing amount of proteins in samples.
