# Supplementary material for: The MsrAB reducing pathway of Streptococcus gordonii is needed for oxidative stress tolerance, biofilm formation, and oral colonization in mice
Source: PLoS One. 2020 Feb 21;15(2):e0229375. doi: 10.1371/journal.pone.0229375 (PMC7034828; doi:10.1371/journal.pone.0229375)
Supplement: S5 Fig — (PDF) [file pone.0229375.s005.pdf]

Fig 3a

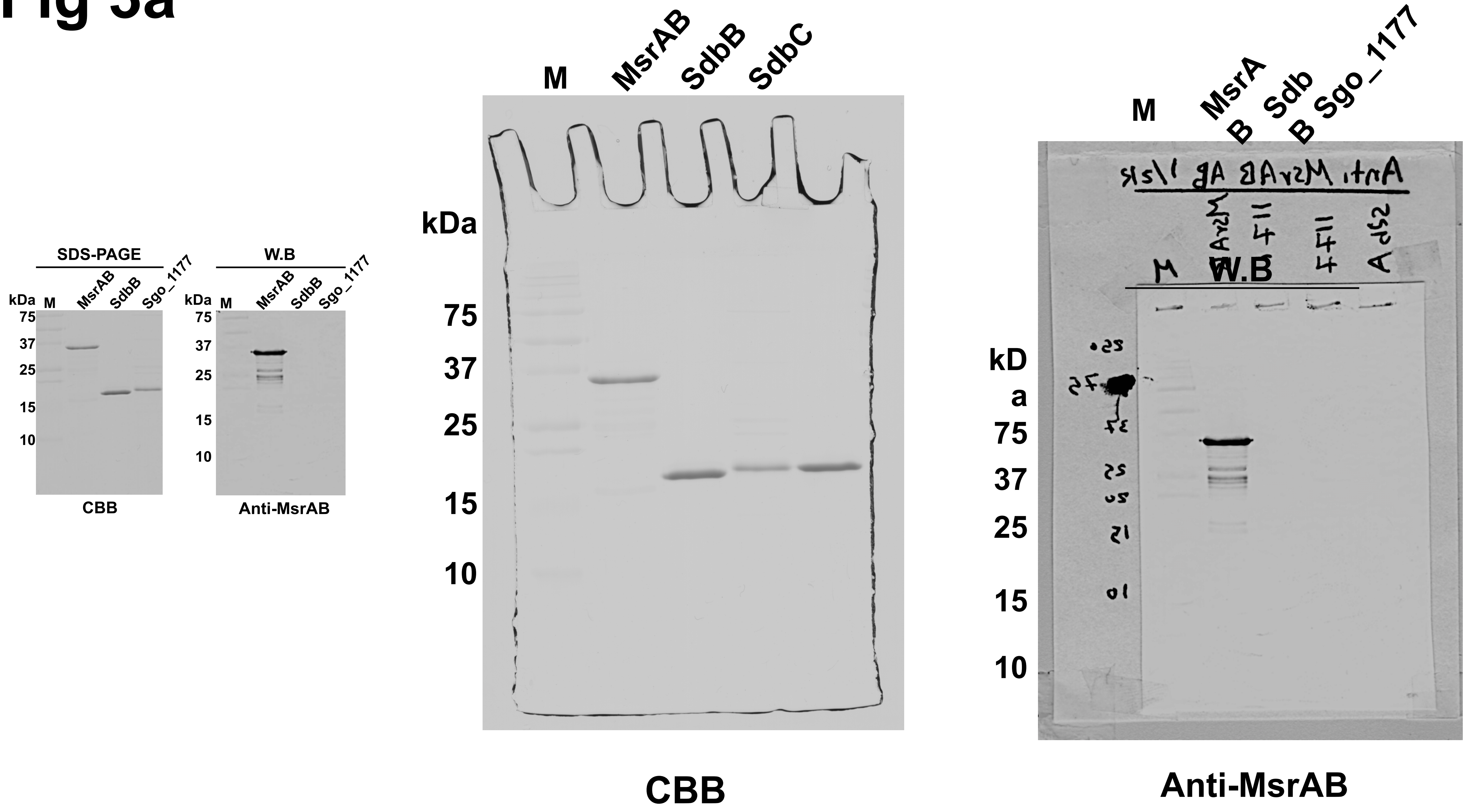
SDS-PAGE
kDa
M
MsrAB
SdbB
Sgo_1177
75
37
25
15
10
CBB
W.B
kDa
M
MsrAB
SdbB
Sgo_1177
75
37
25
15
10
Anti-MsrAB
M
MsrAB
SdbB
SdbC
kDa
75
37
25
15
10
CBB
M
MsrA B
Sdb B
Sgo_1177
W.B
kD a
75
37
25
15
10
Anti-MsrAB

Fig 3b and c

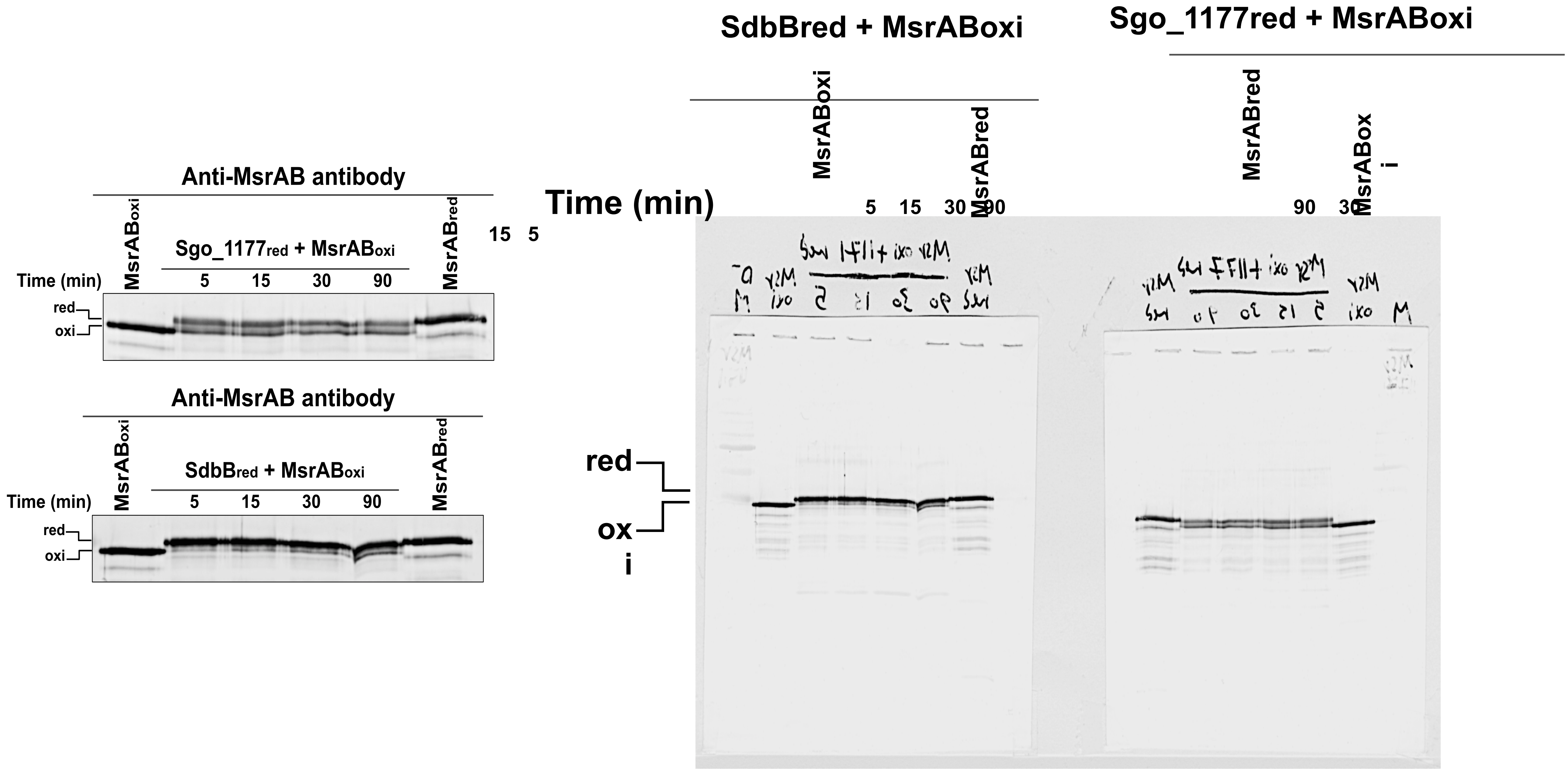

Anti-MsrAB antibody
MsrABoxi
Sgo_1177red + MsrABoxi
MsrABred
Time (min)
5
15
30
90
red
oxi
Anti-MsrAB antibody
MsrABoxi
SdbBred + MsrABoxi
MsrABred
Time (min)
5
15
30
90
red
oxi
15 5
SdbBred + MsrABoxi
Sgo_1177red + MsrABoxi
MsrABoxi
MsrABred
MsrABred
MsrABox
i
Time (min)
5 15 30 90
90 30
red
ox
i

Fig 3d

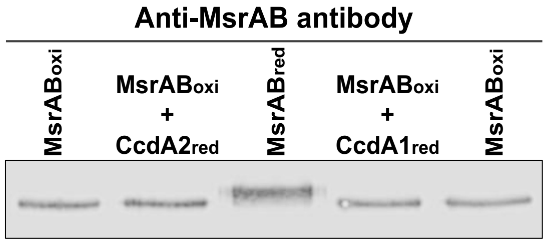
Anti-MsrAB antibody
MsrABoxi
MsrABoxi + CcdA2red
MsrABred
MsrABoxi + CcdA1red
MsrABoxi

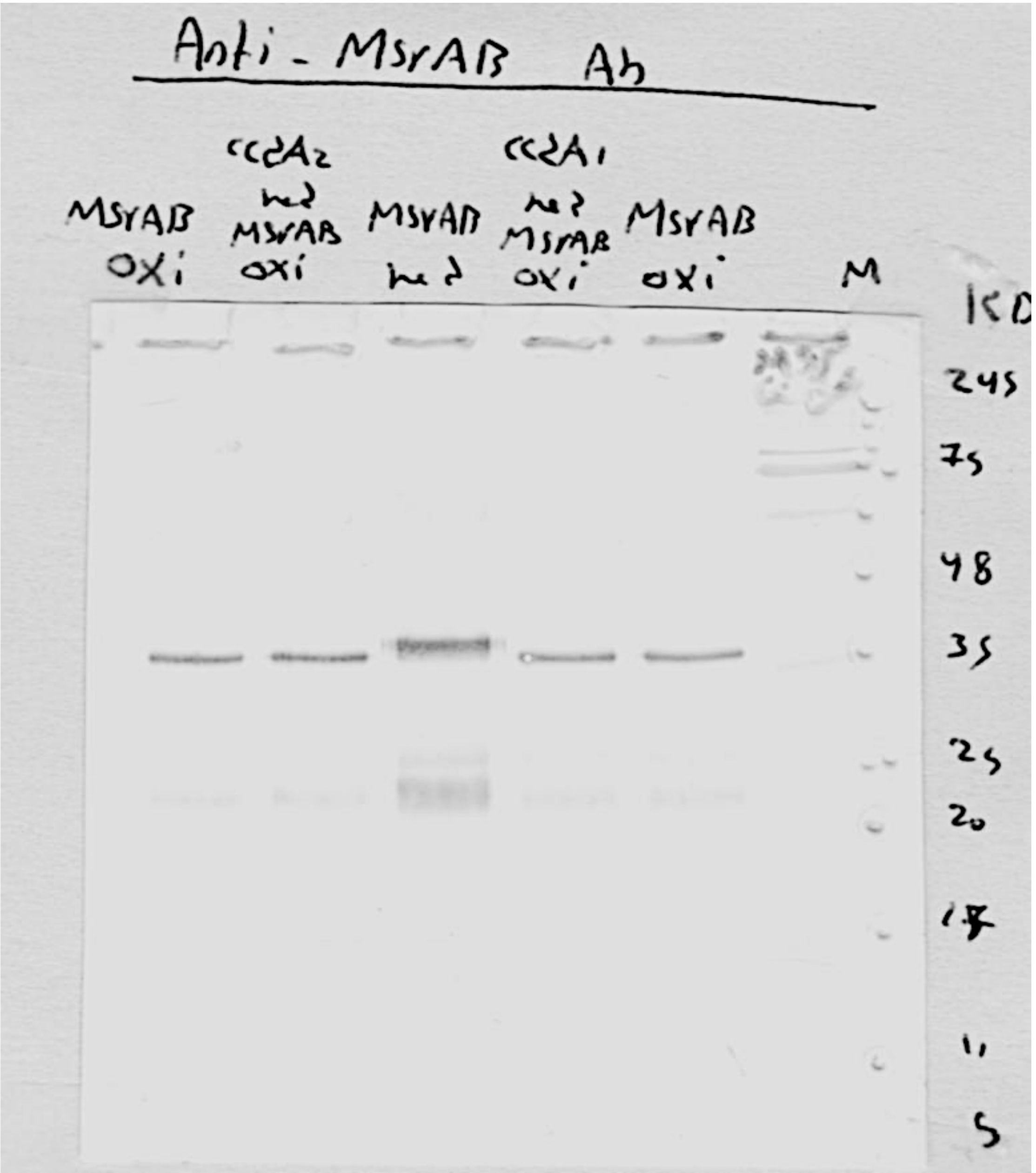
Anti - MsrAB Ab
MsrAB oxi
CcdA2 red MsrAB oxi
MsrAB red
CcdA1 red MsrAB oxi
MsrAB oxi
M
KD
245
75
48
35
25
20
17
11
5

Fig 3e

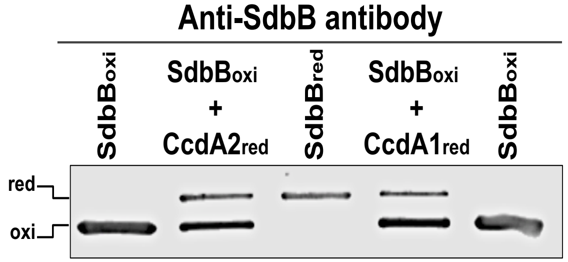
Anti-SdbB antibody
SdbBoxi
SdbBoxi + CcdA2red
SdbBred
SdbBoxi + CcdA1red
SdbBoxi
red
oxi

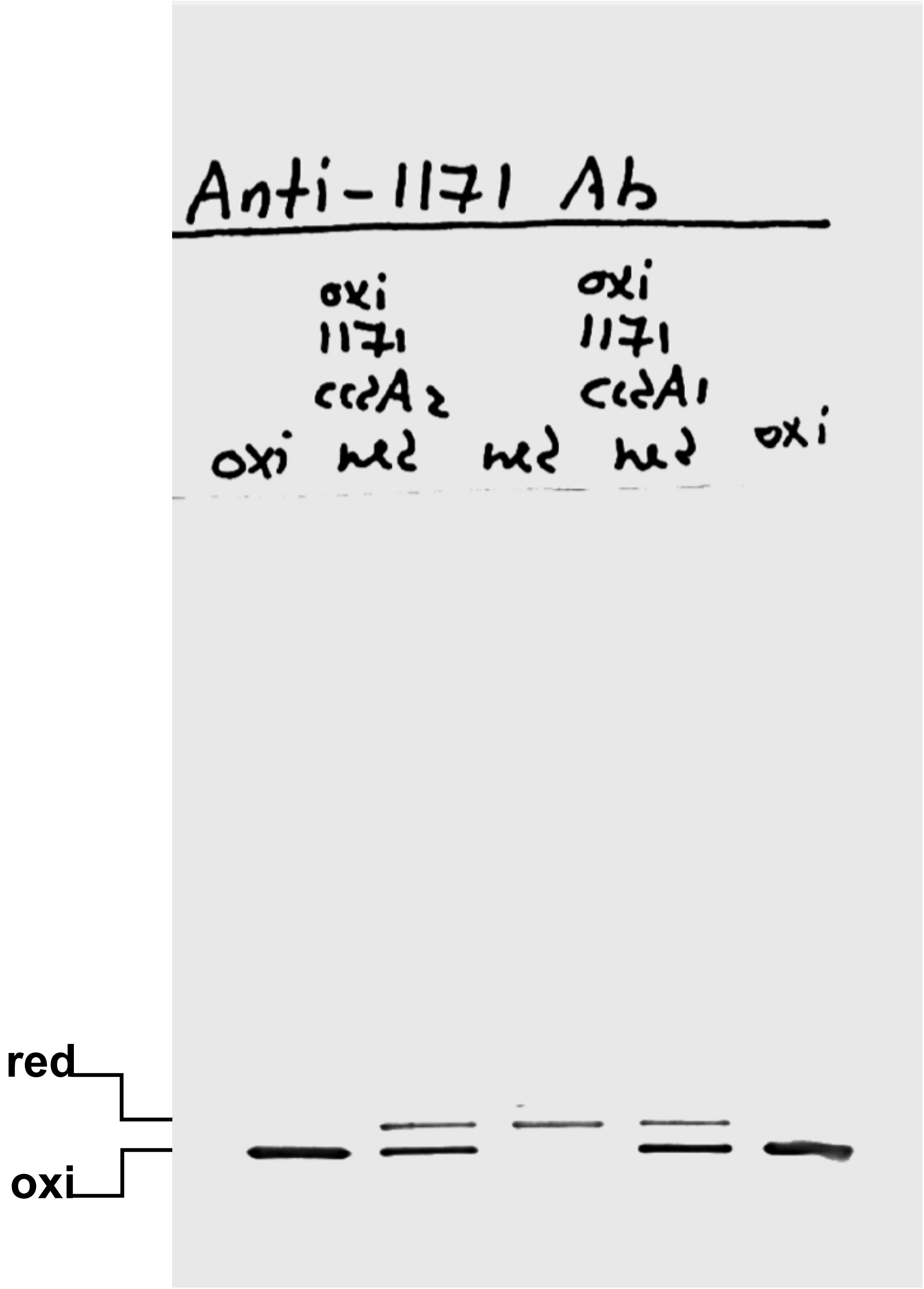
Anti-1171 Ab
oxi 1171 ccdA2
oxi 1171 ccdA1
oxi
red
red
red
oxi
red
oxi

Fig 3f

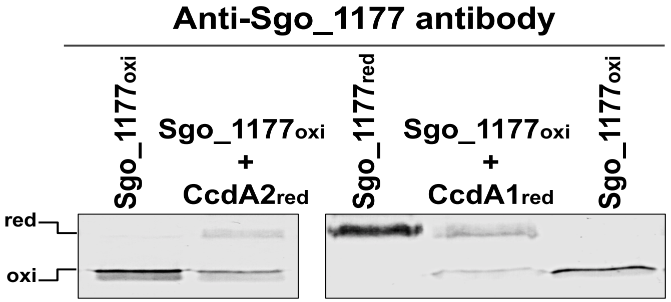

Anti-Sgo_1177 antibody
Sgo_1177oxi
Sgo_1177oxi + CcdA2red
Sgo_1177red
Sgo_1177oxi + CcdA1red
Sgo_1177oxi
red
oxi

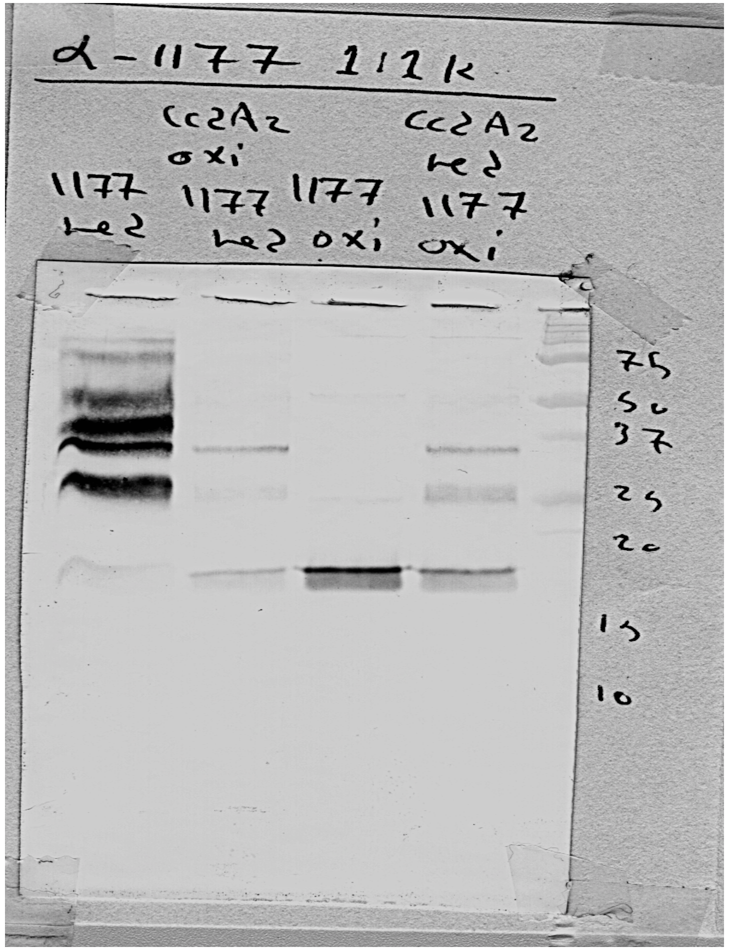

α-1177 1:1k
ccdA2 oxi
ccdA2 red
1177 red
1177 red
1177 oxi
1177 oxi
75
50
37
25
20
15
10

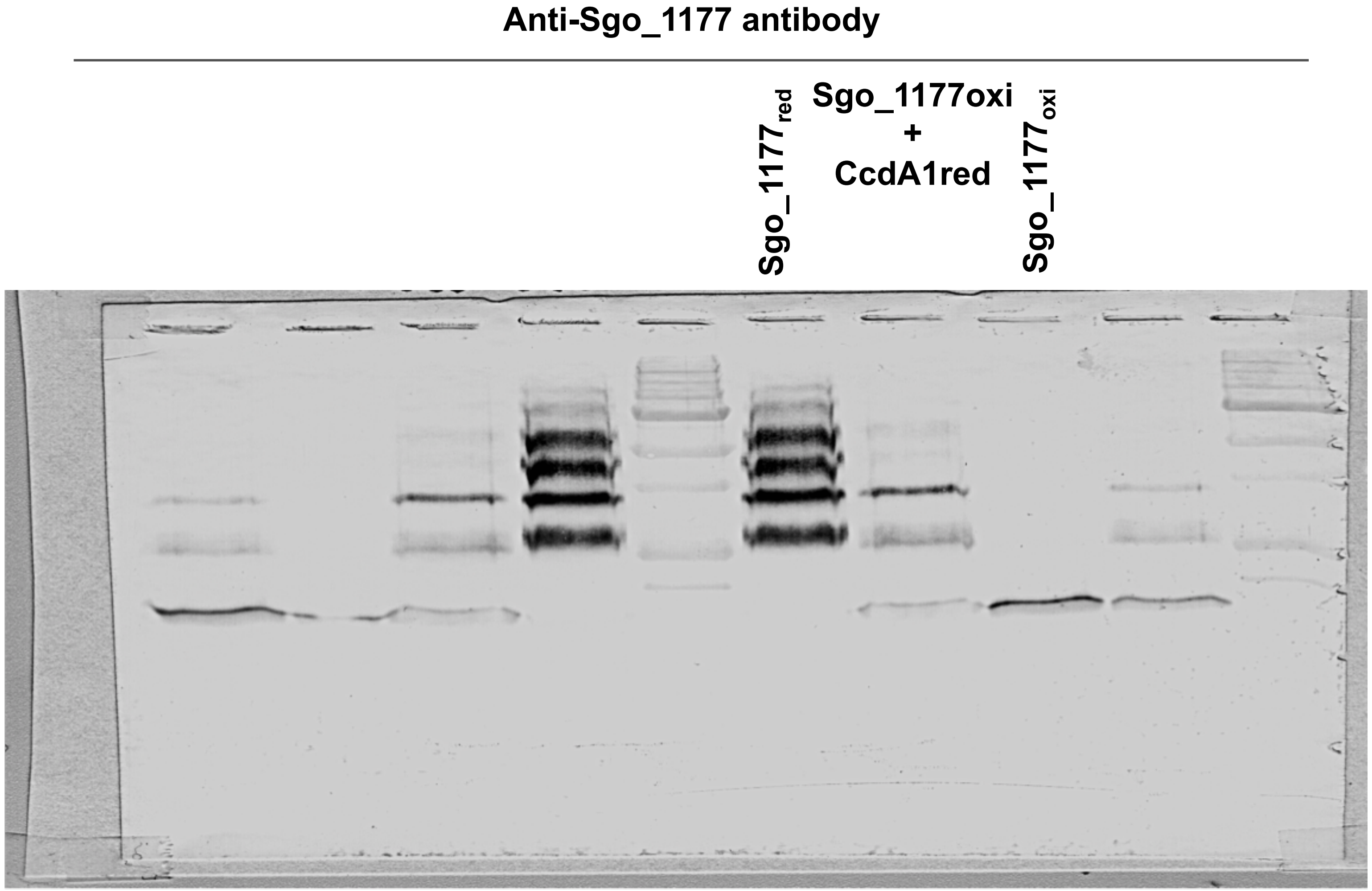

Anti-Sgo_1177 antibody
Sgo_1177red
Sgo_1177oxi + CcdA1red
Sgo_1177oxi

Fig 4b

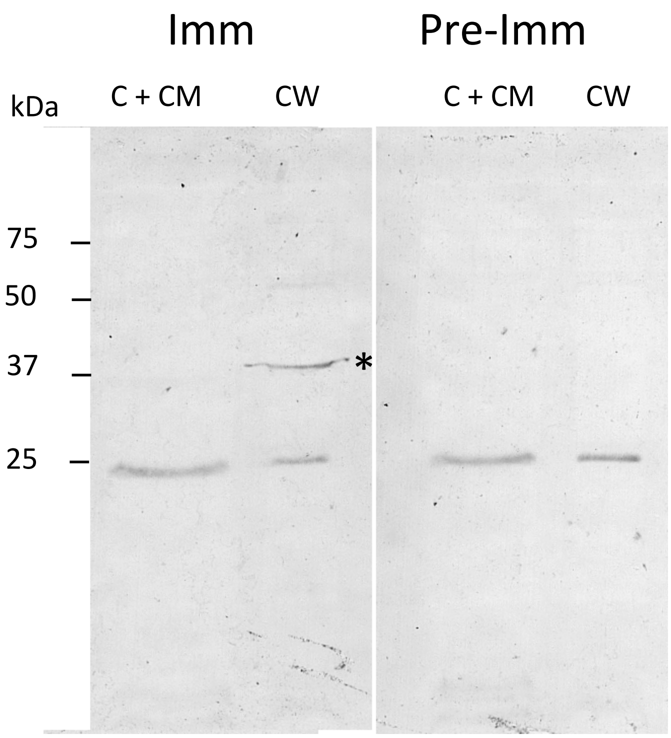

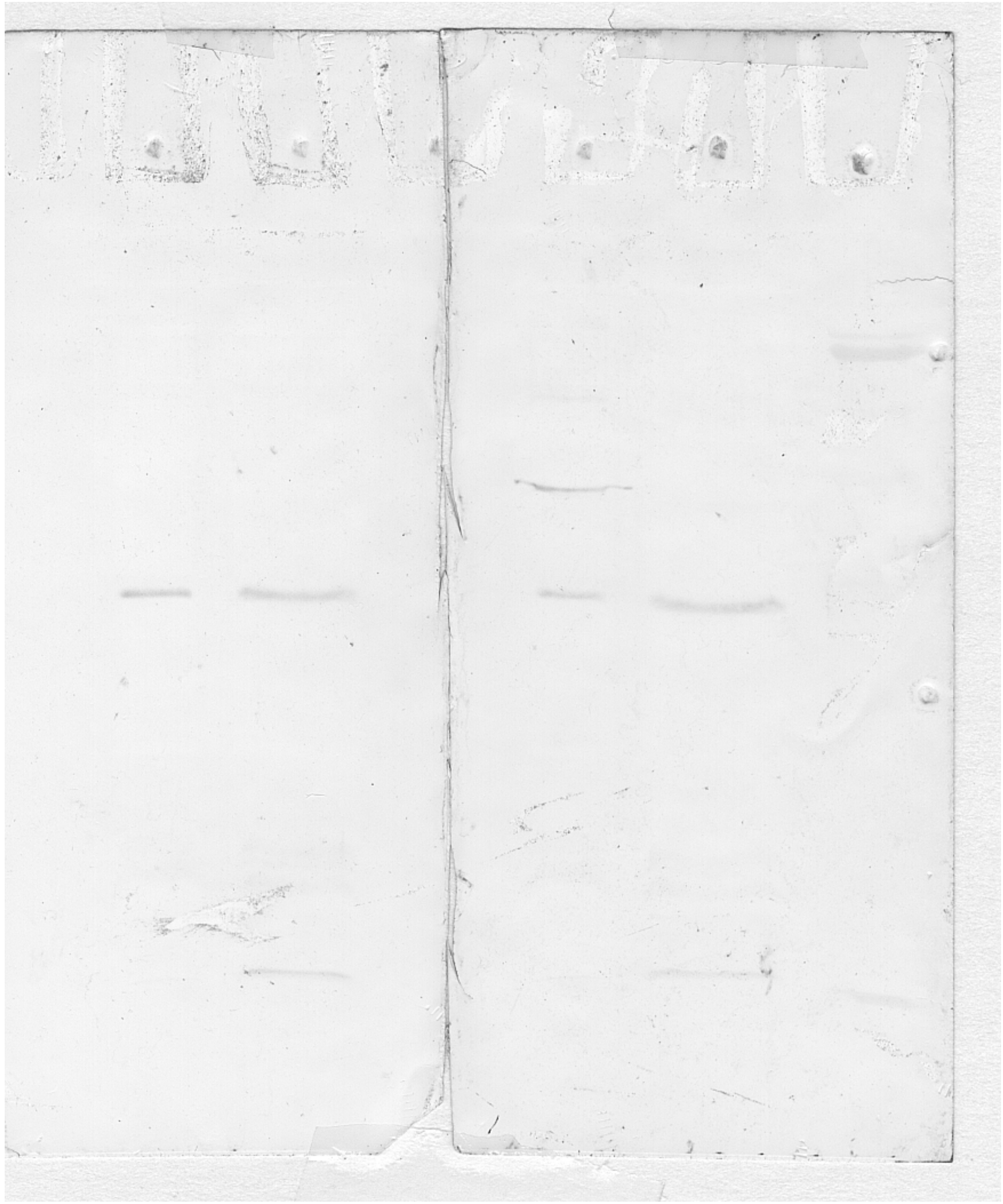

Fig 6a

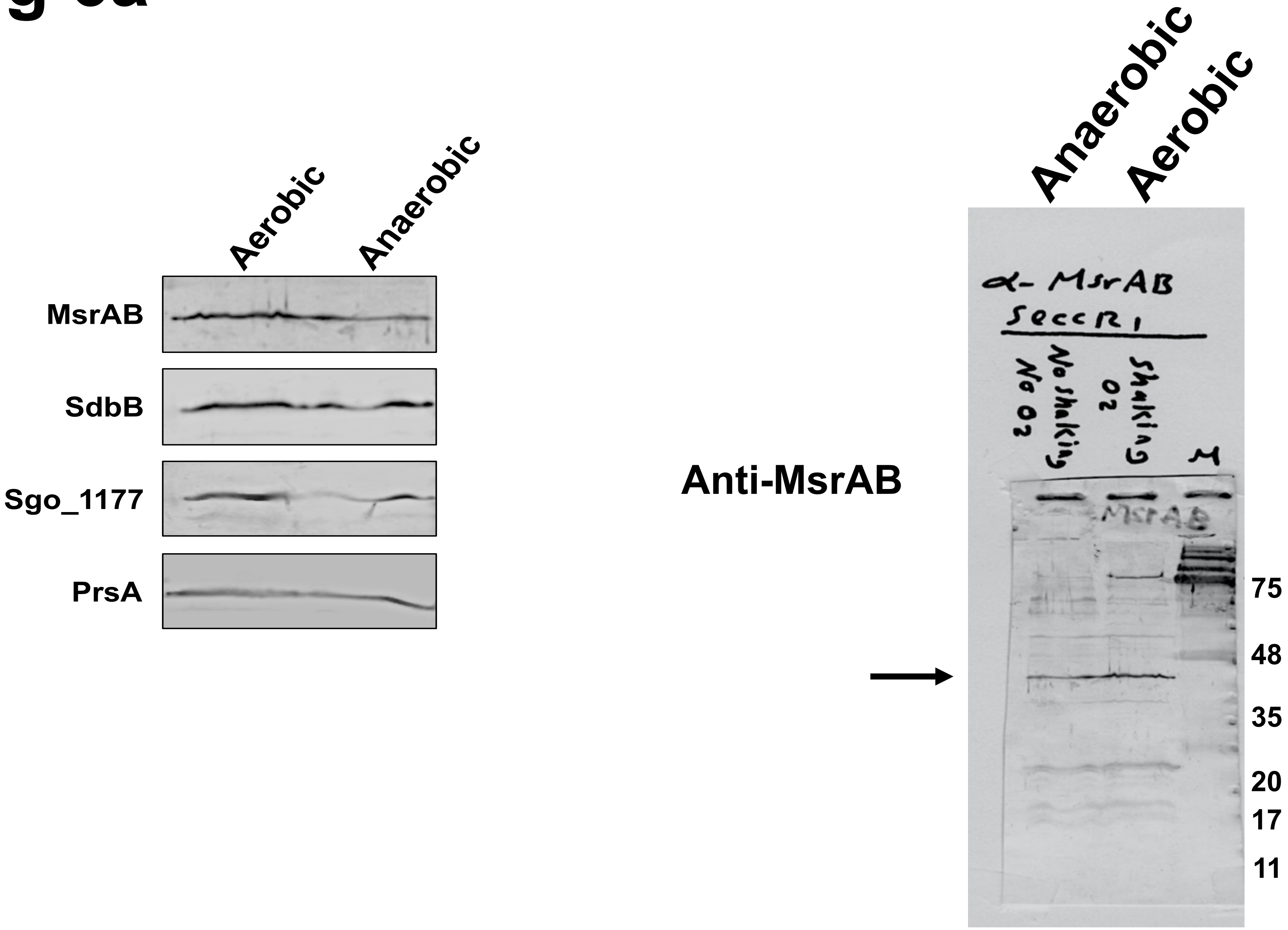
Aerobic
Anaerobic
MsrAB
SdbB
Sgo_1177
PrsA
Anaerobic
Aerobic
Anti-MsrAB
α- MsrAB
No O2
No shaking
Shaking
O2
M
MsrAB
75
48
35
20
17
11

Fig 6a

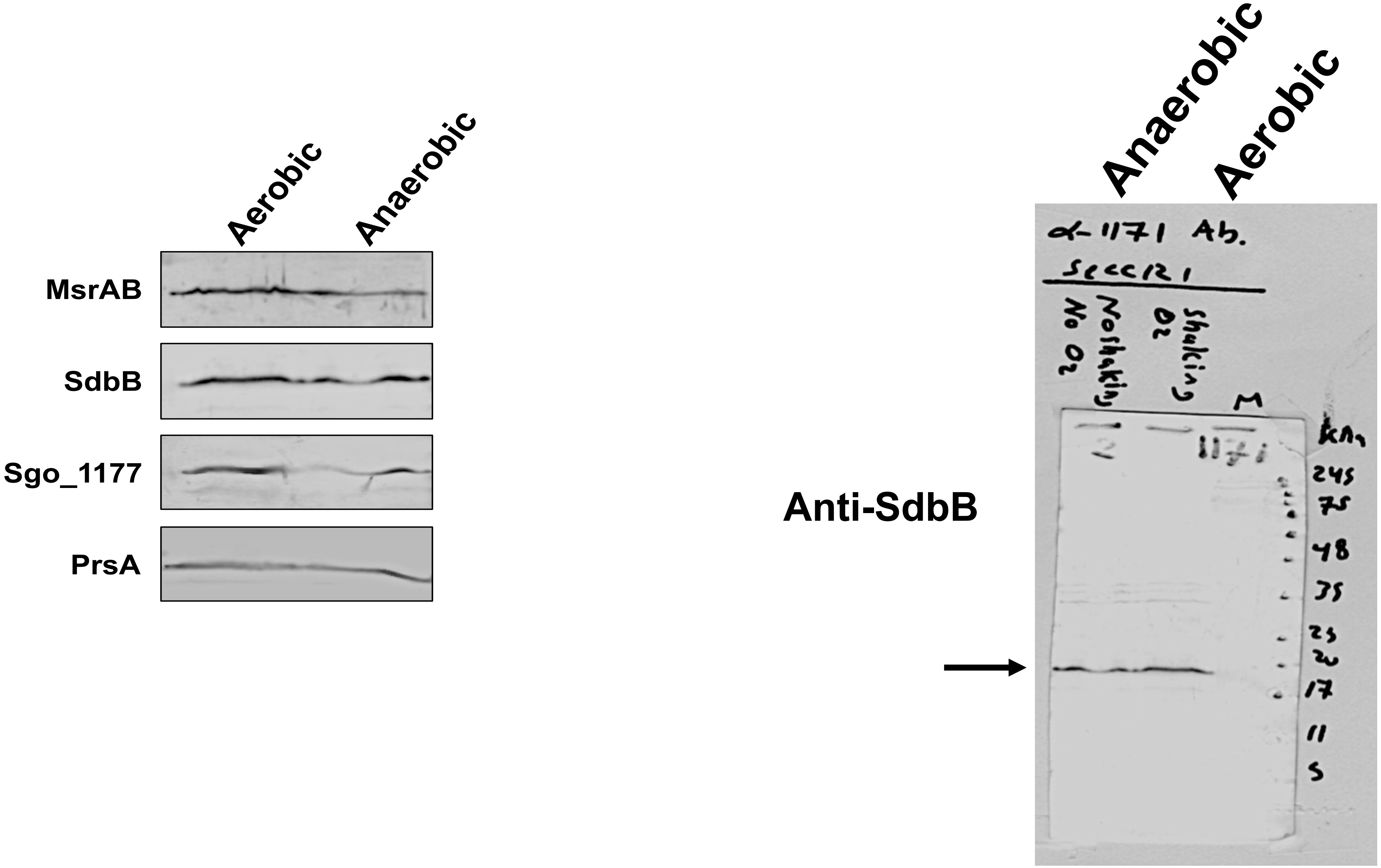
Aerobic
Anaerobic
MsrAB
SdbB
Sgo_1177
PrsA
Anaerobic
Aerobic
Anti-SdbB

Fig 6a

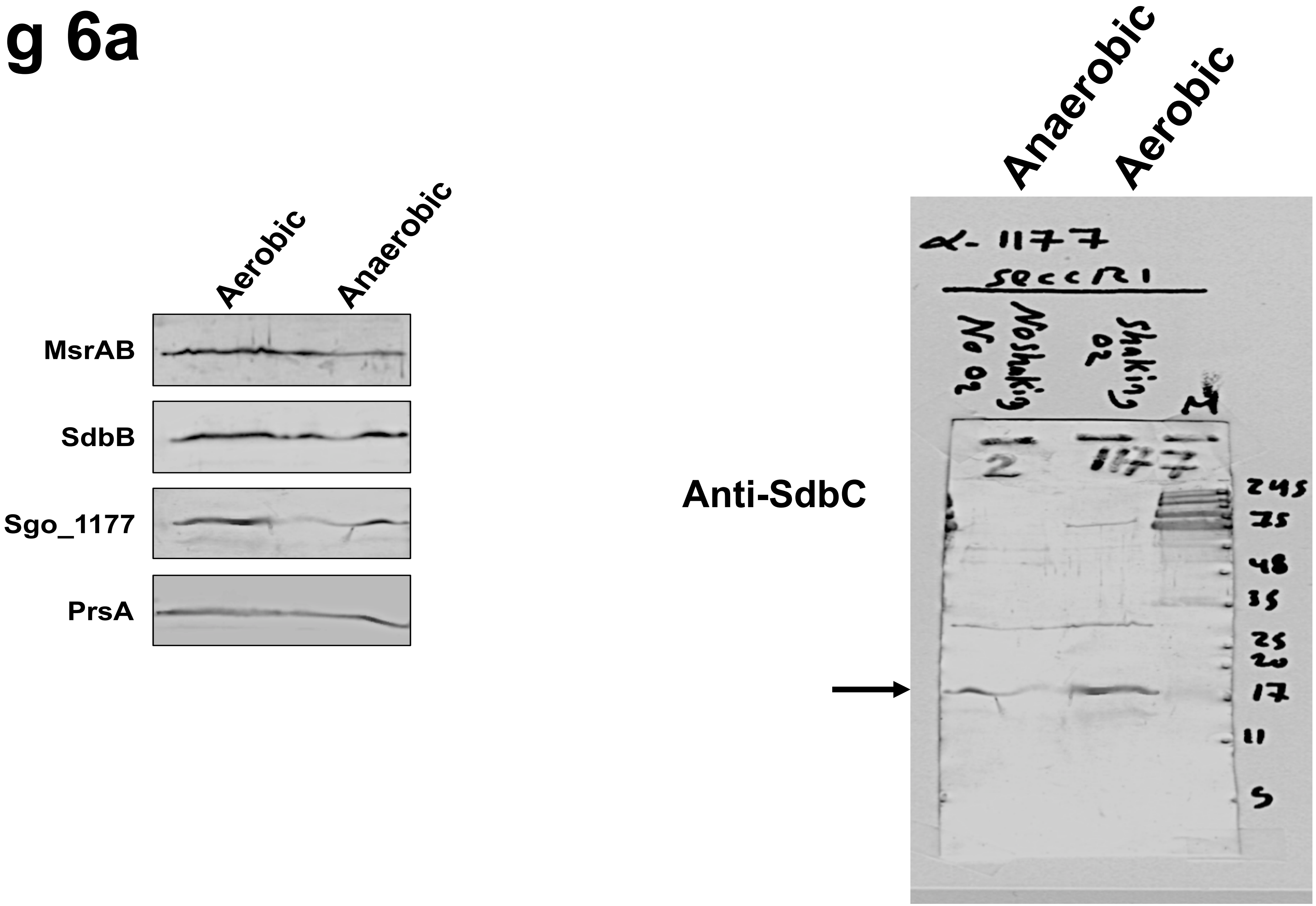
Aerobic
Anaerobic
MsrAB
SdbB
Sgo_1177
PrsA
Anaerobic
Aerobic
Anti-SdbC
245
75
48
35
25
20
17
11
5

Fig 6a

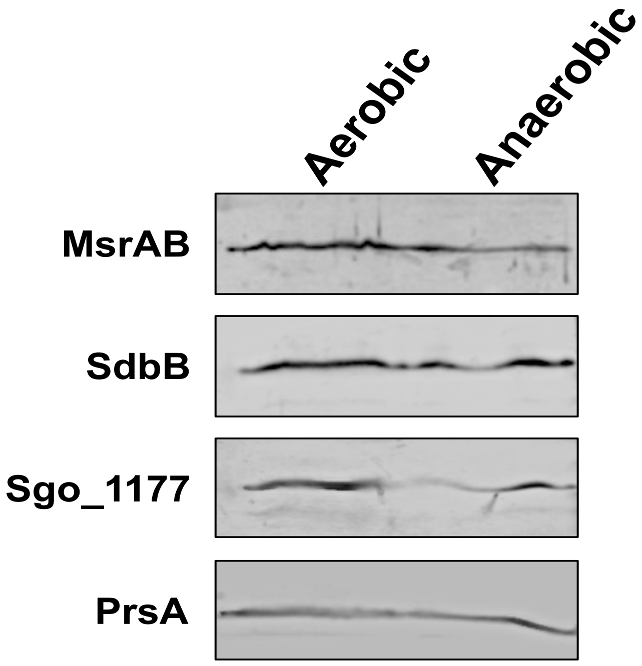
Aerobic
Anaerobic
MsrAB
SdbB
Sgo_1177
PrsA

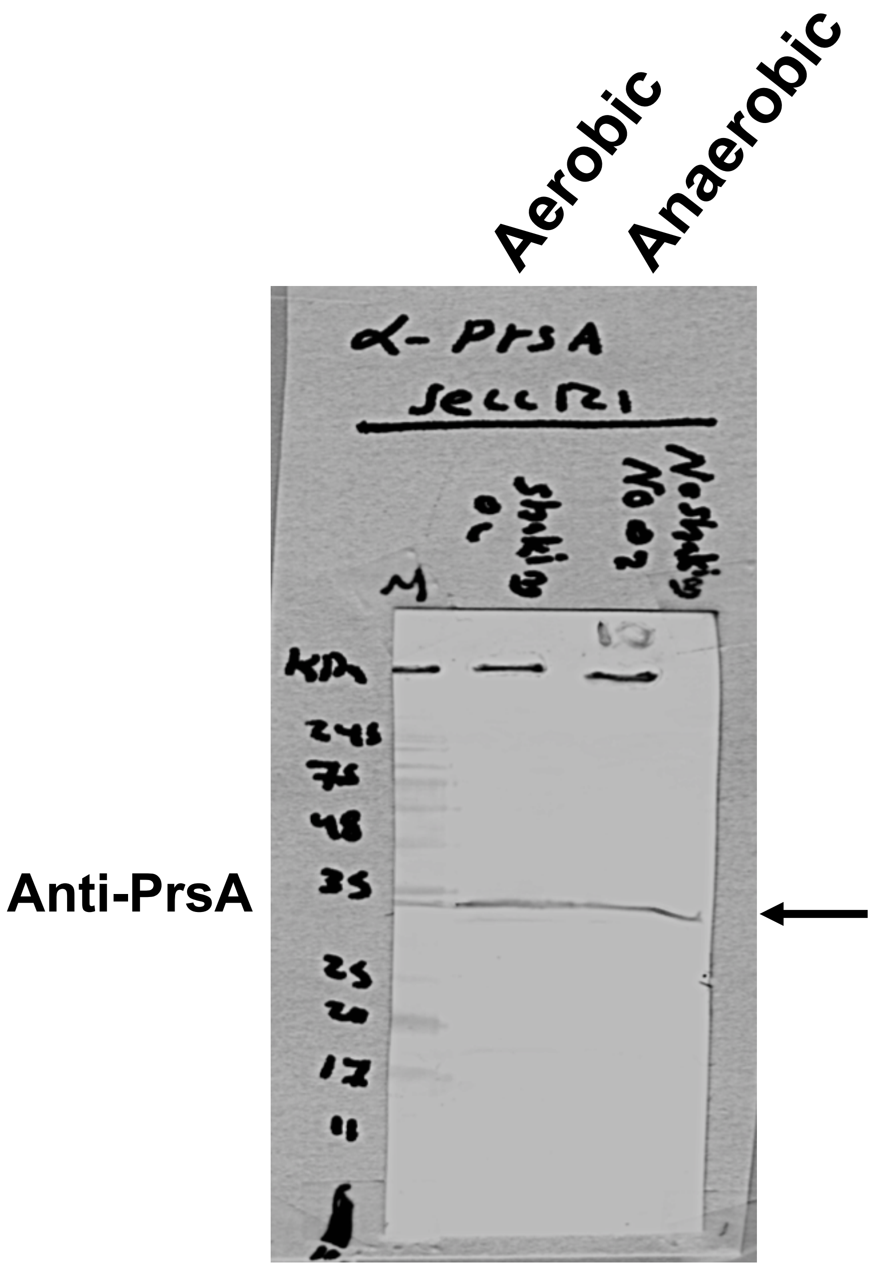
Aerobic
Anaerobic
Anti-PrsA

Fig 6b

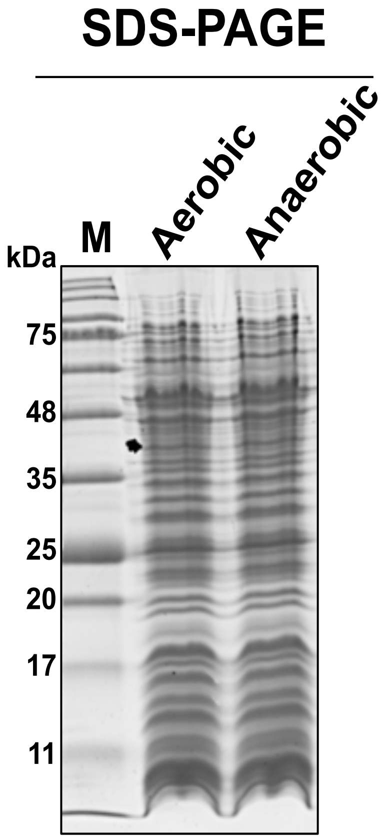
SDS-PAGE
M
Aerobic
Anaerobic
kDa
75
48
35
25
20
17
11

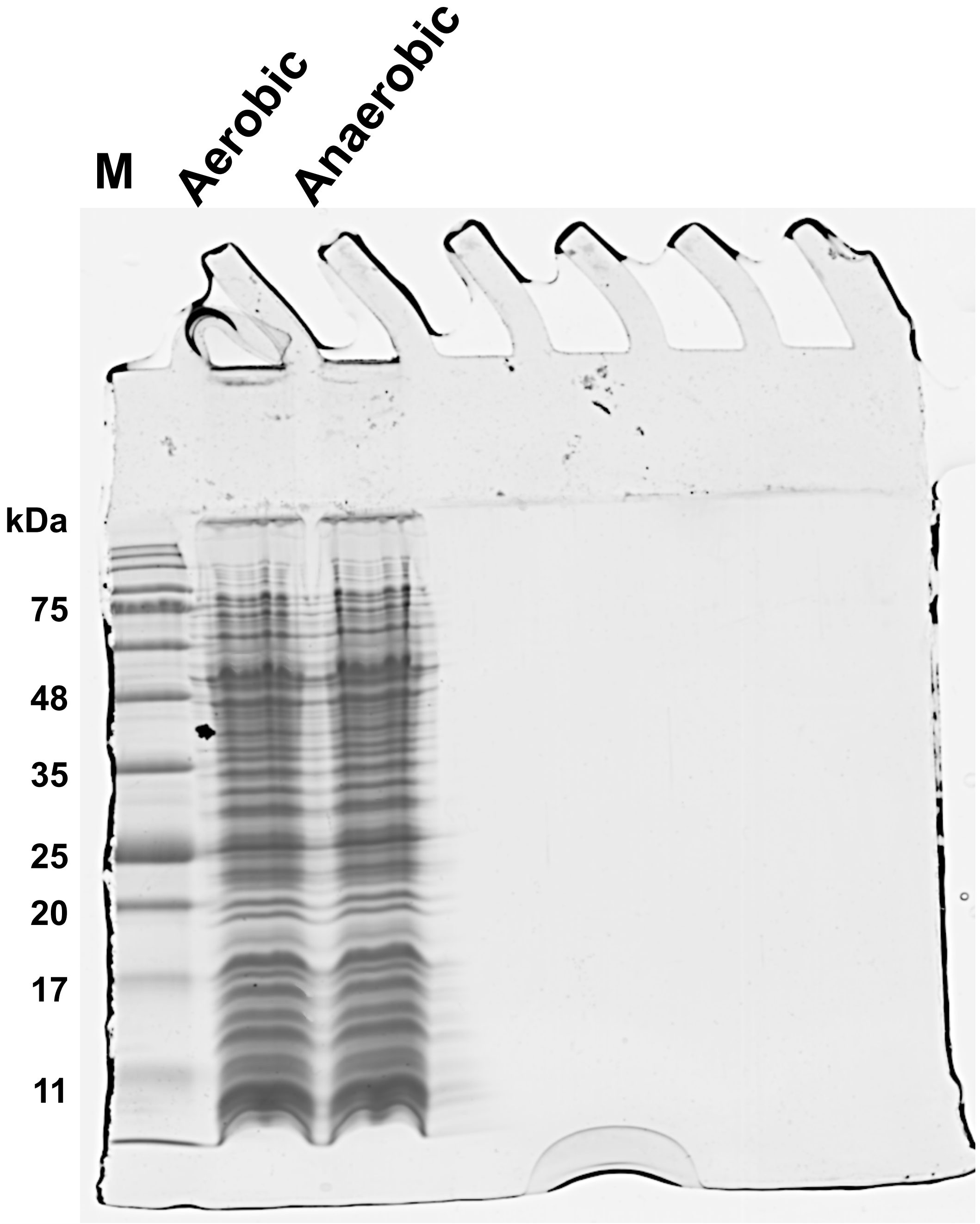
M
Aerobic
Anaerobic
kDa
75
48
35
25
20
17
11

Fig 6d

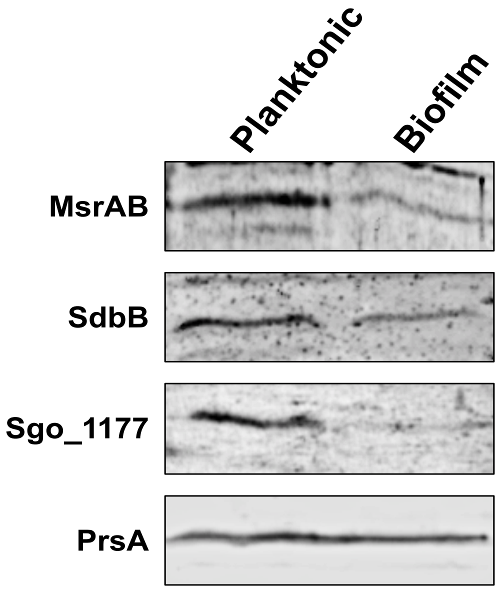

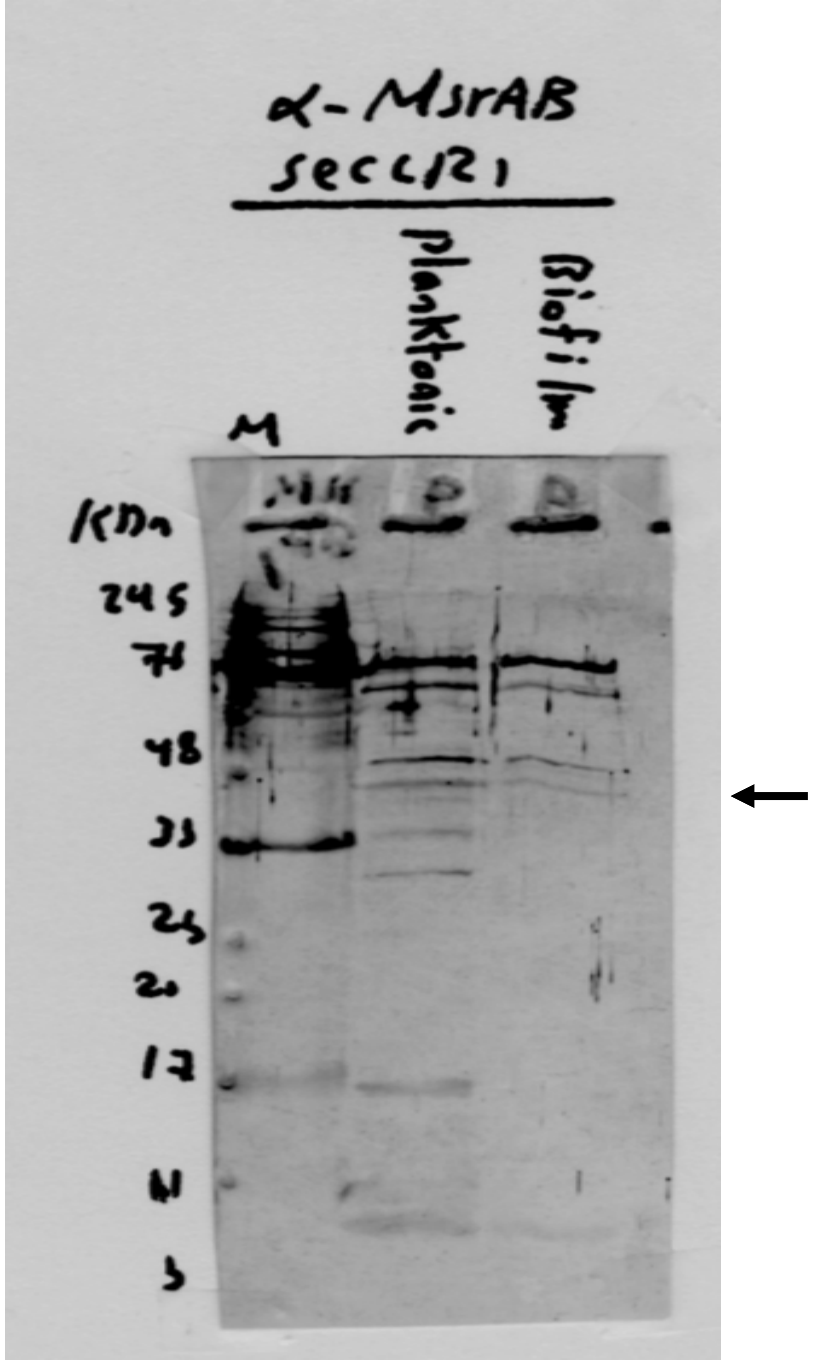

Anti-MsrAB

Fig 6d

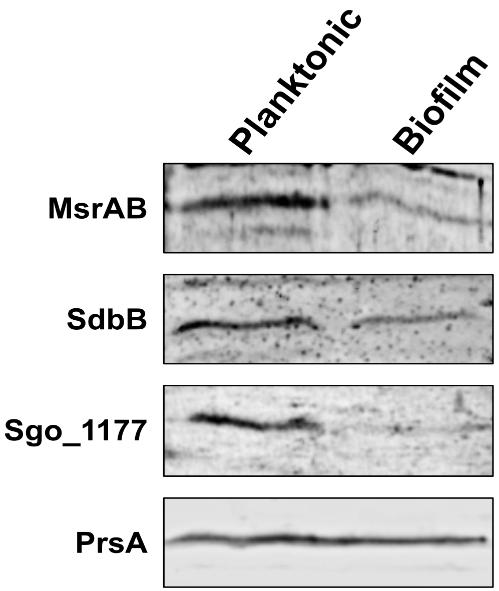
Planktonic
Biofilm
MsrAB
SdbB
Sgo_1177
PrsA

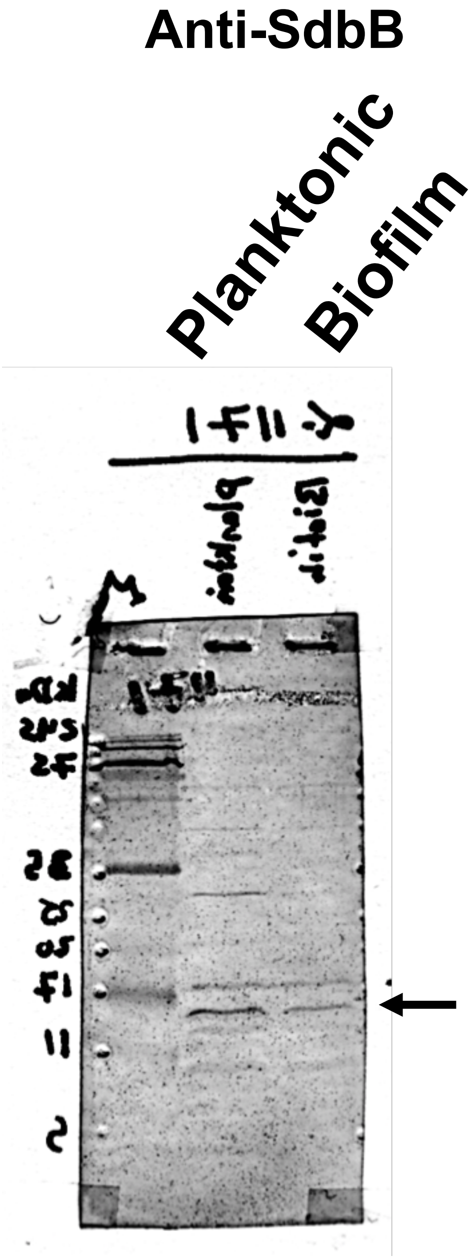
Anti-SdbB
Planktonic
Biofilm

Fig 6d

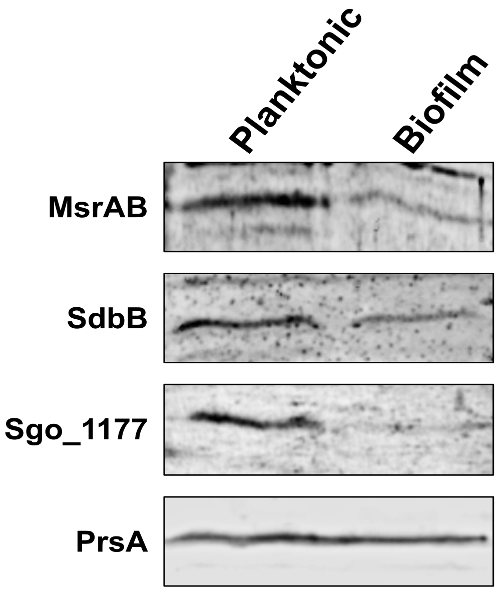

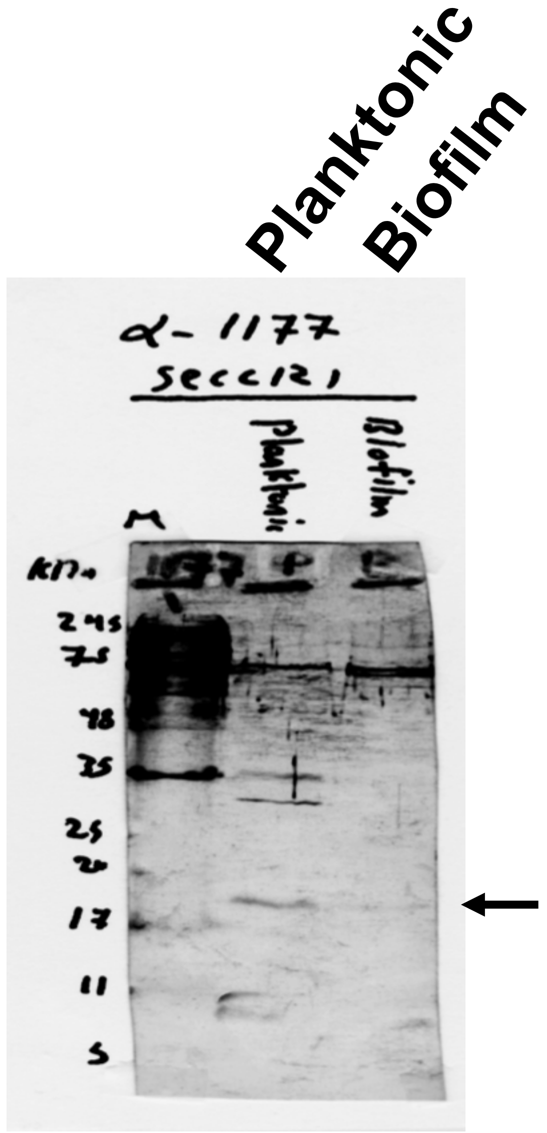

Anti-Sgo_1177

Fig 6d

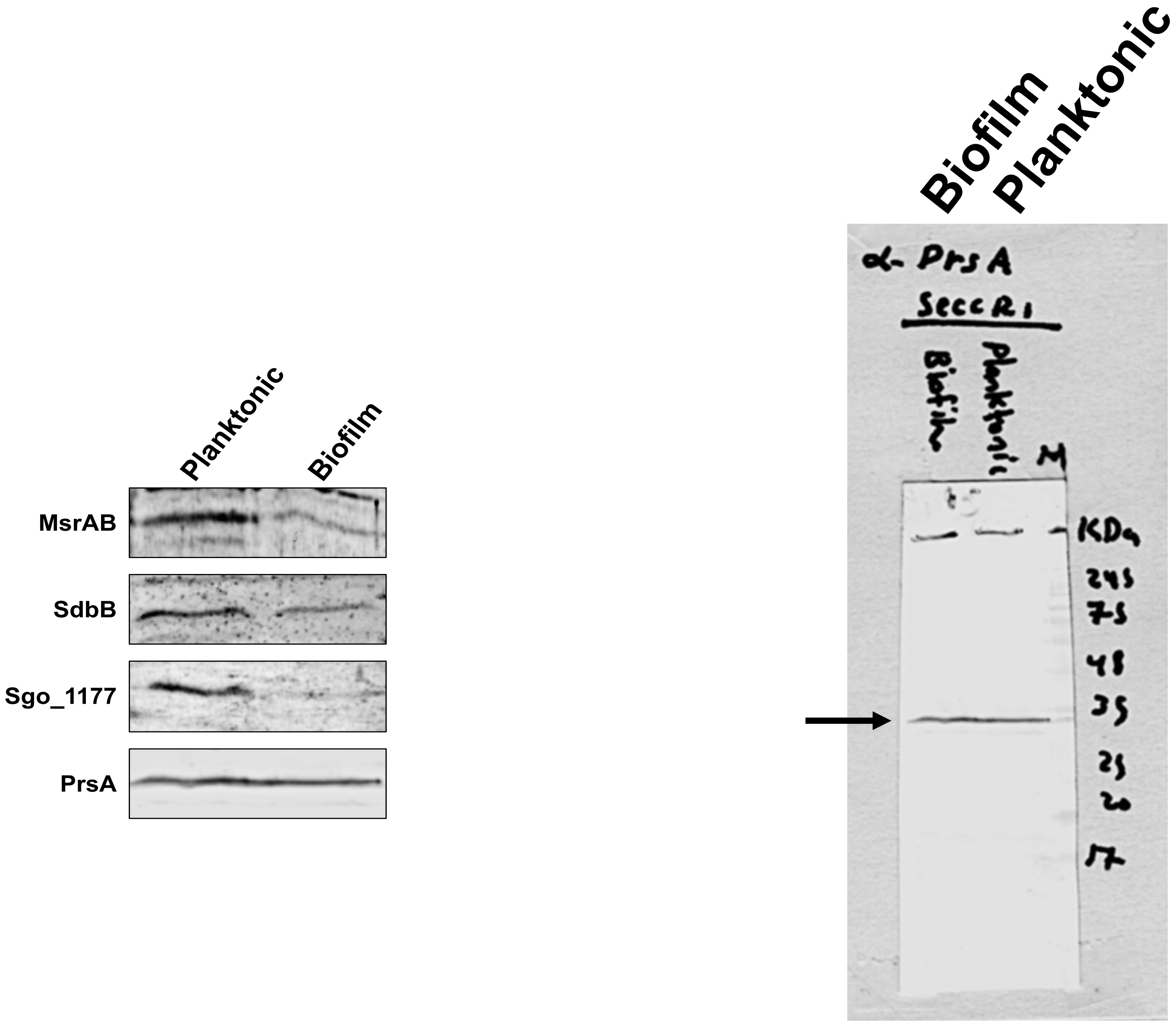

Anti-PrsA

Fig 6e

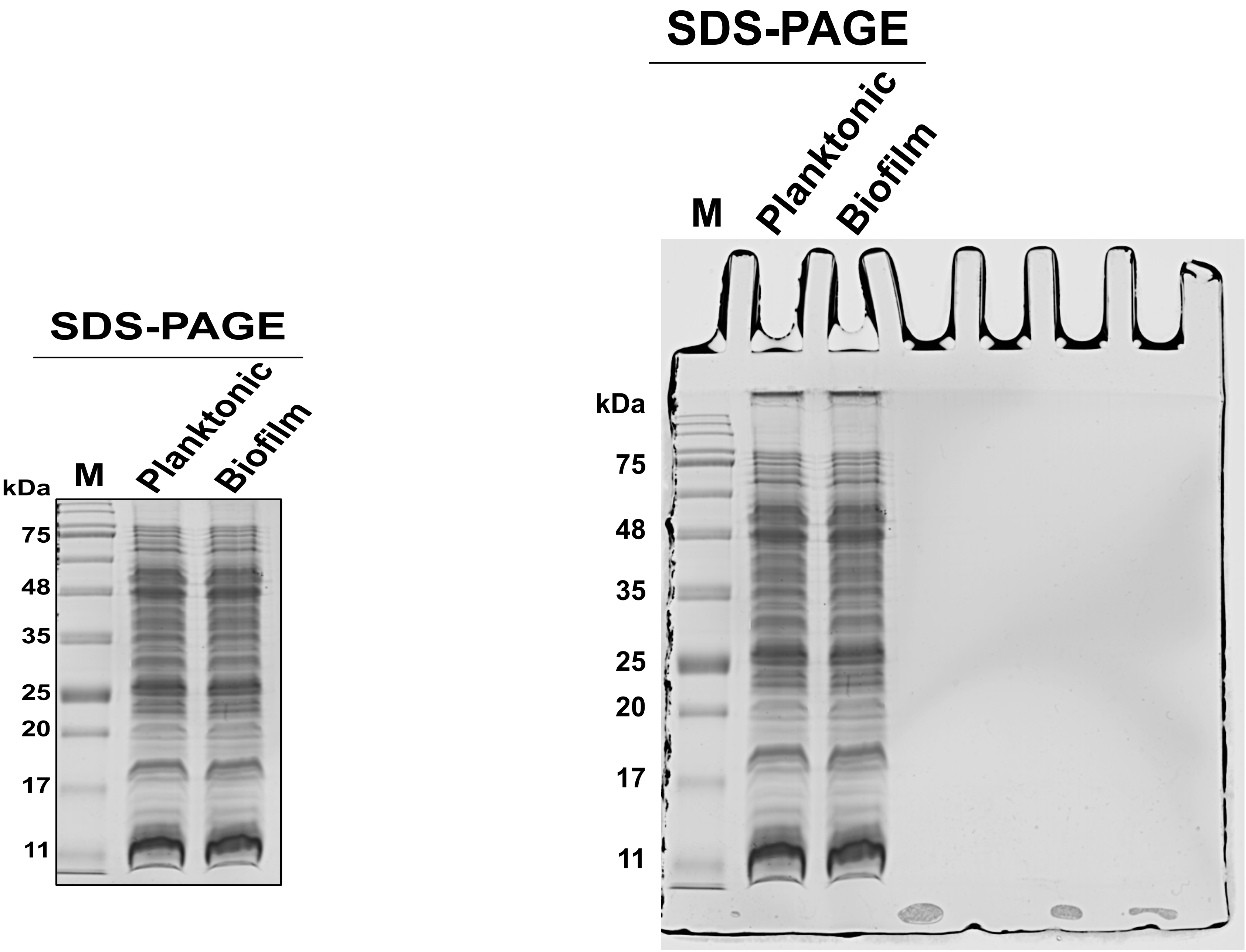
SDS-PAGE
M
Planktonic
Biofilm
kDa
75
48
35
25
20
17
11
SDS-PAGE
M
Planktonic
Biofilm
kDa
75
48
35
25
20
17
11

S2 Fig

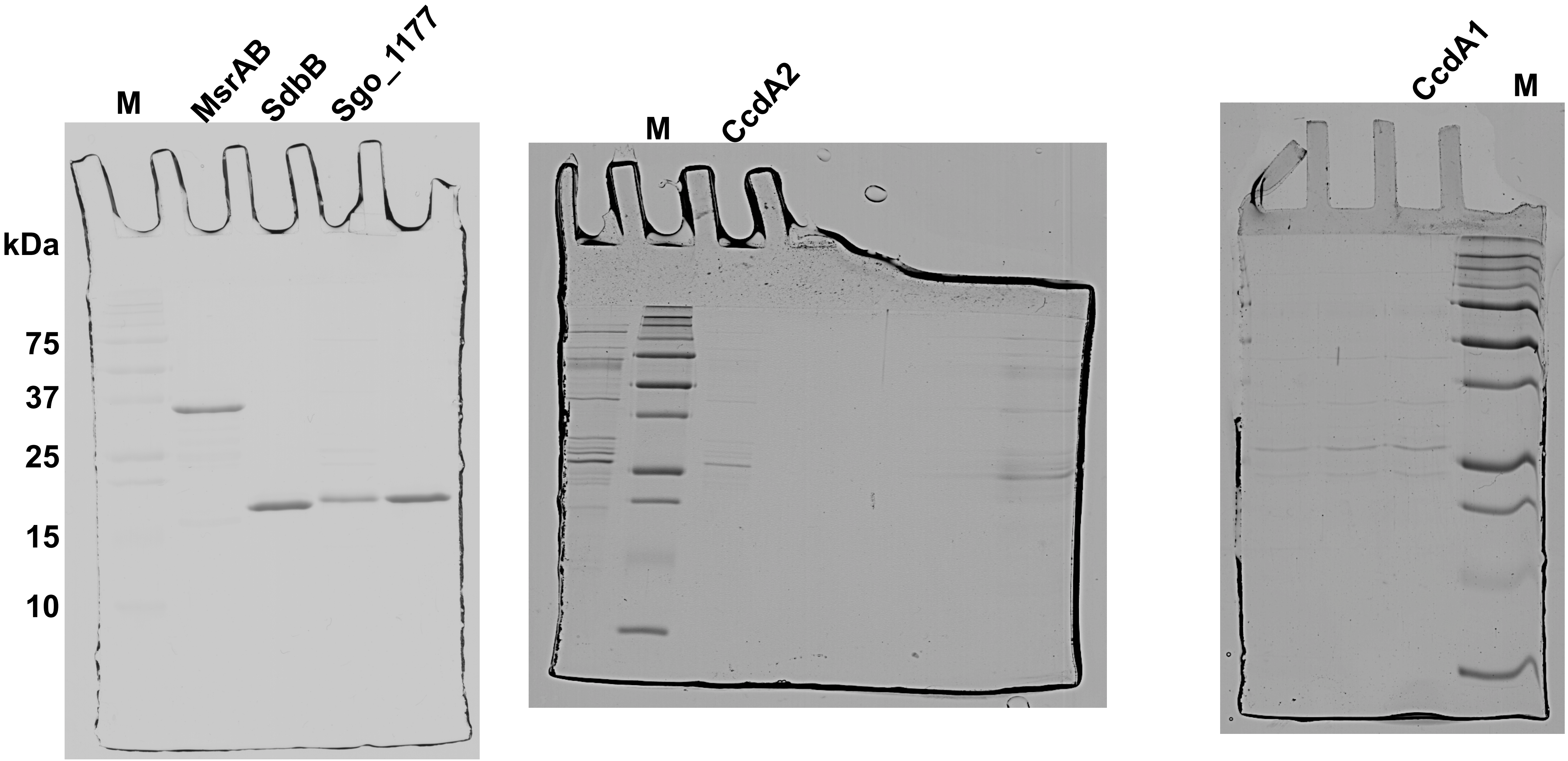

S2 Fig

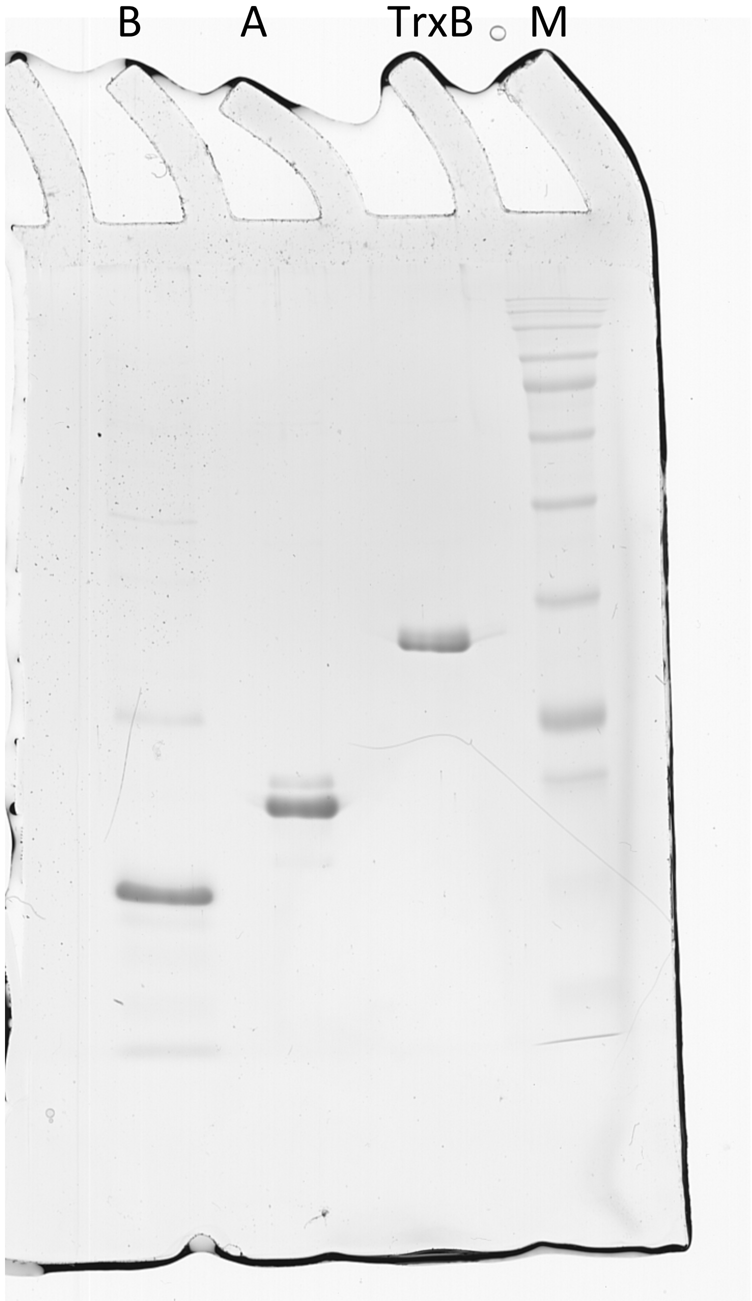

S4 Fig

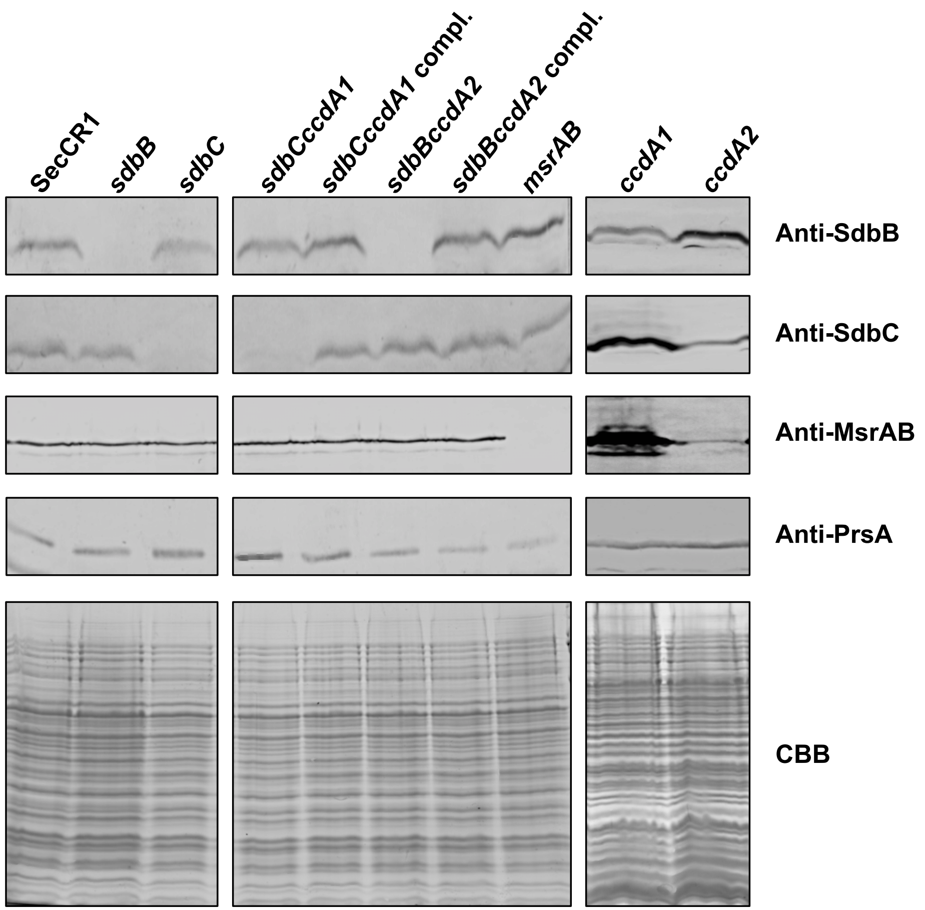

SecCR1
sdbB
sdbC
sdbCccdA1
sdbCccdA1 compl.
sdbBccdA2
sdbBccdA2 compl.
msrAB
ccdA1
ccdA2
Anti-SdbB
Anti-SdbC
Anti-MsrAB
Anti-PrsA
CBB

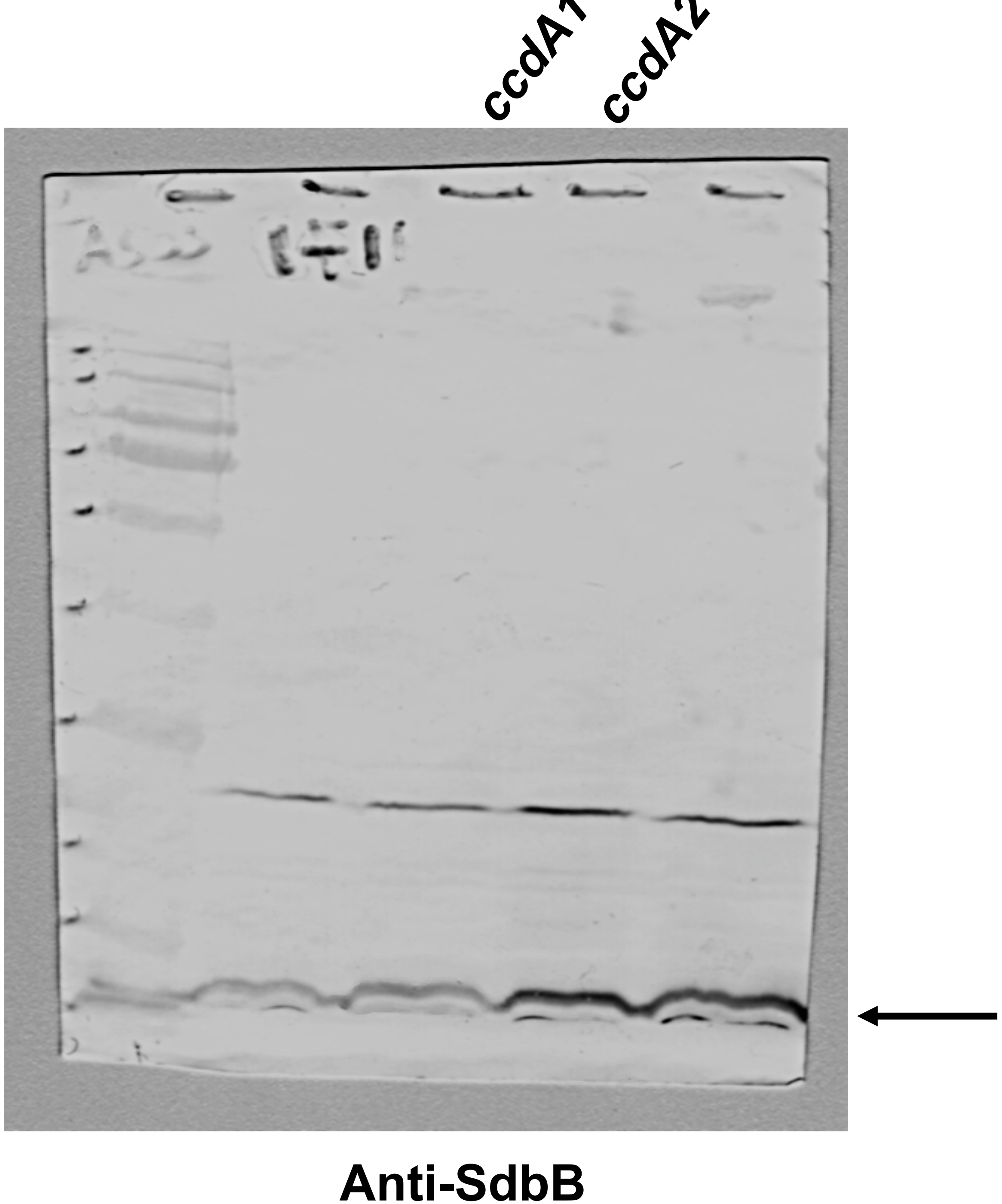

ccdA1
ccdA2
Anti-SdbB

S4 Fig

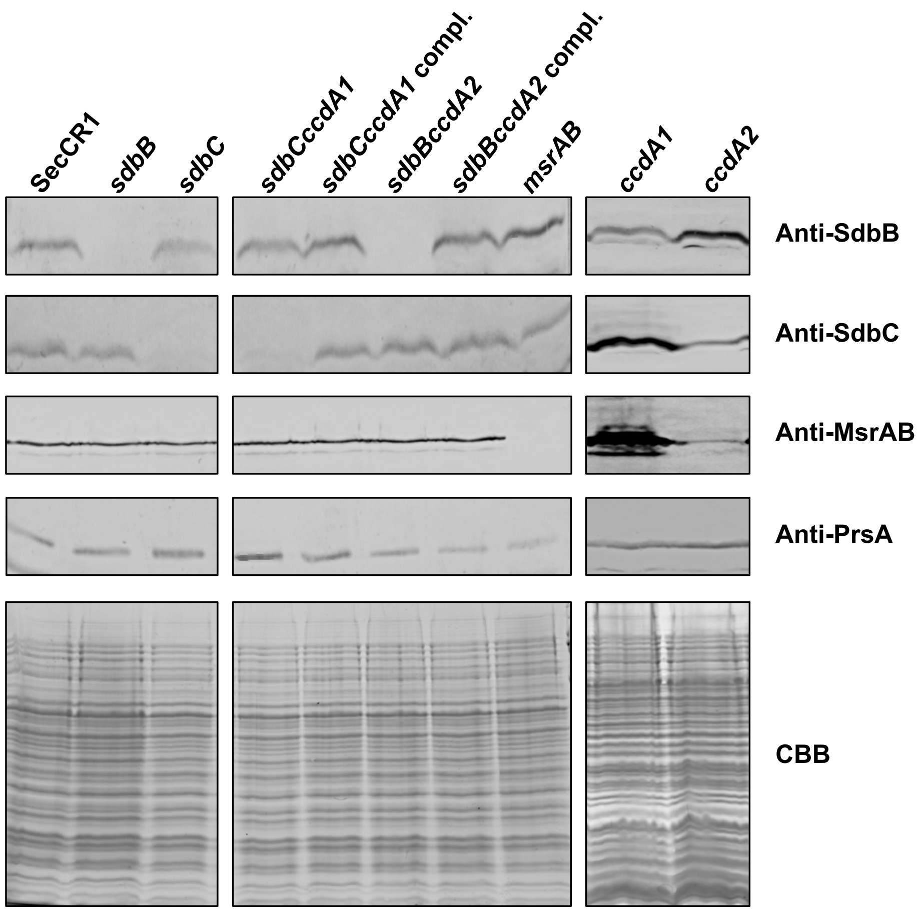

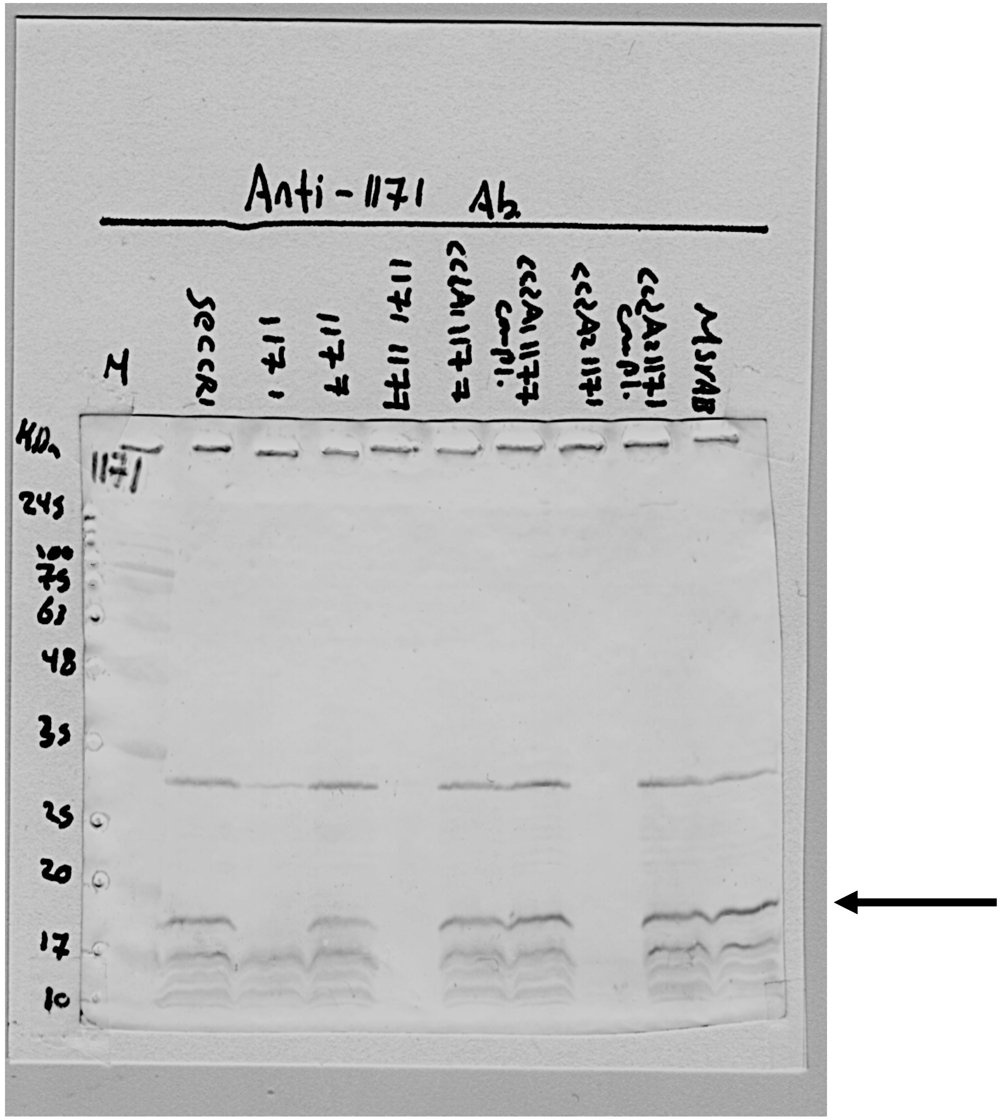

Anti-SdbB

S4 Fig

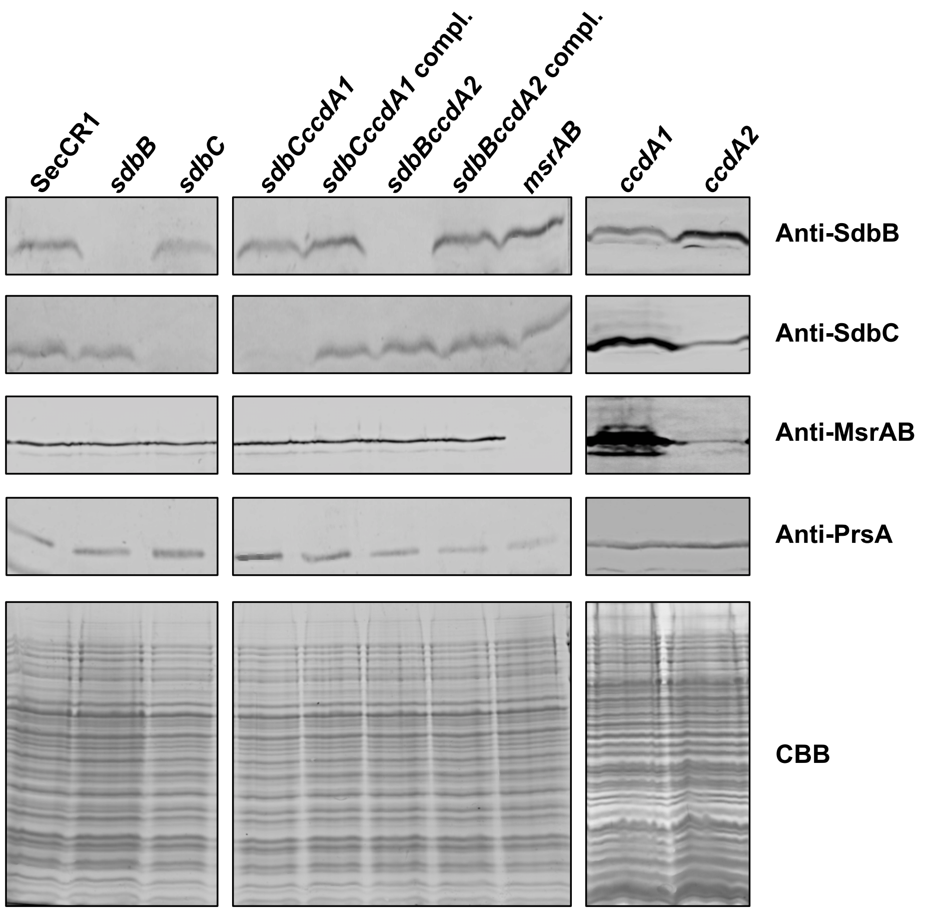
SecCR1
sdbB
sdbC
sdbCccdA1
sdbCccdA1 compl.
sdbBccdA2
sdbBccdA2 compl.
msrAB
ccdA1
ccdA2
Anti-SdbB
Anti-SdbC
Anti-MsrAB
Anti-PrsA
CBB

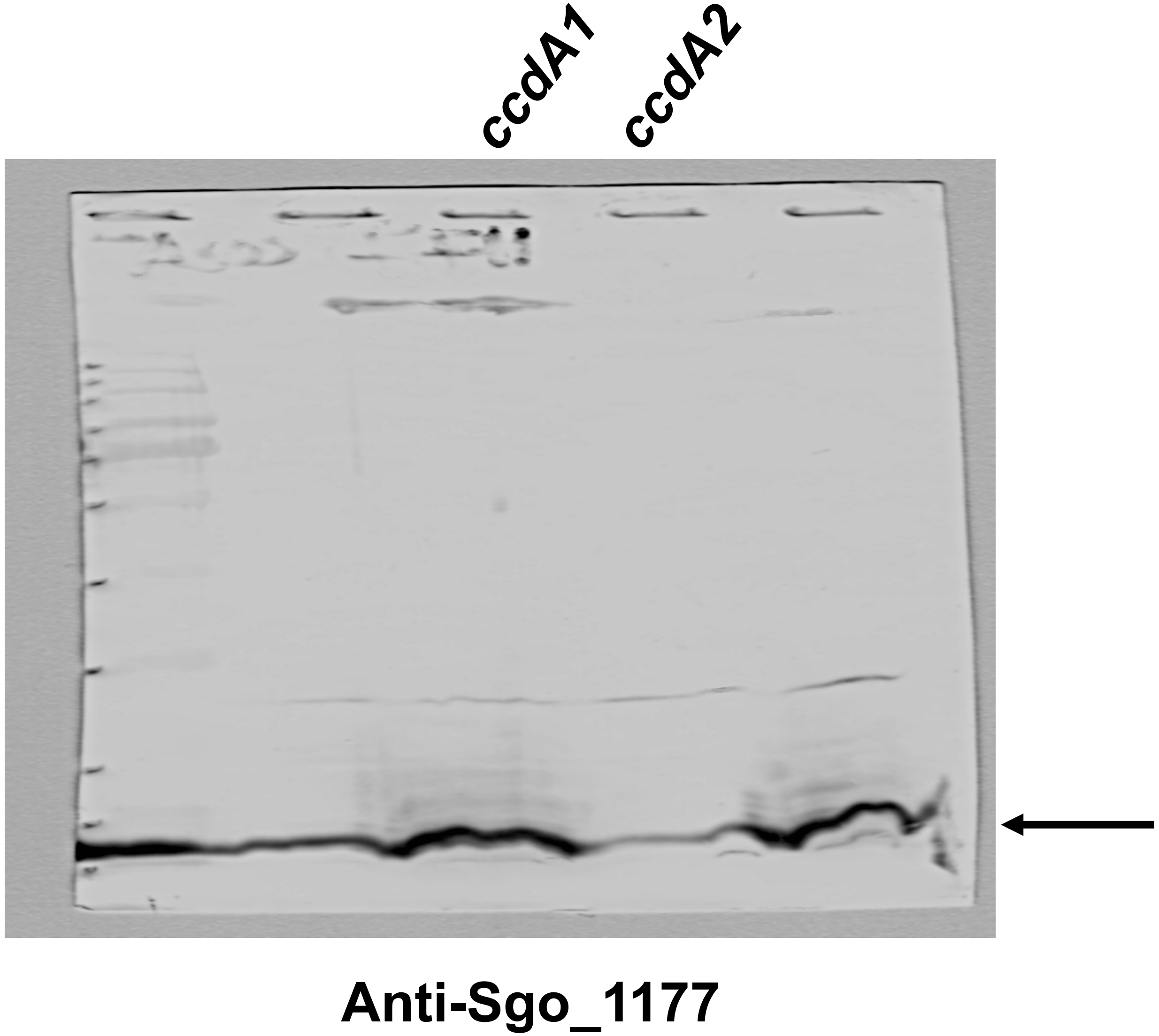
ccdA1
ccdA2
Anti-Sgo_1177

S4 Fig

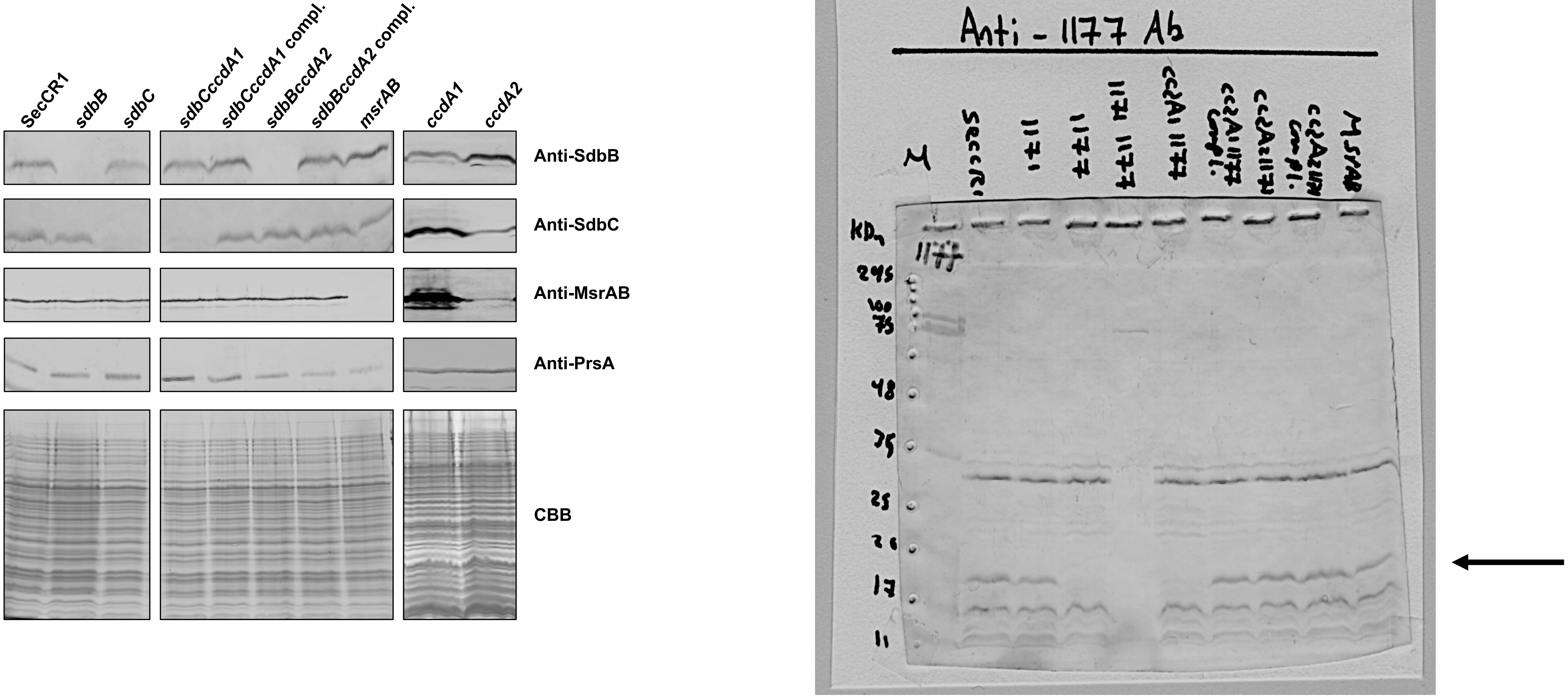

Anti-Sgo_1177

S4 Fig

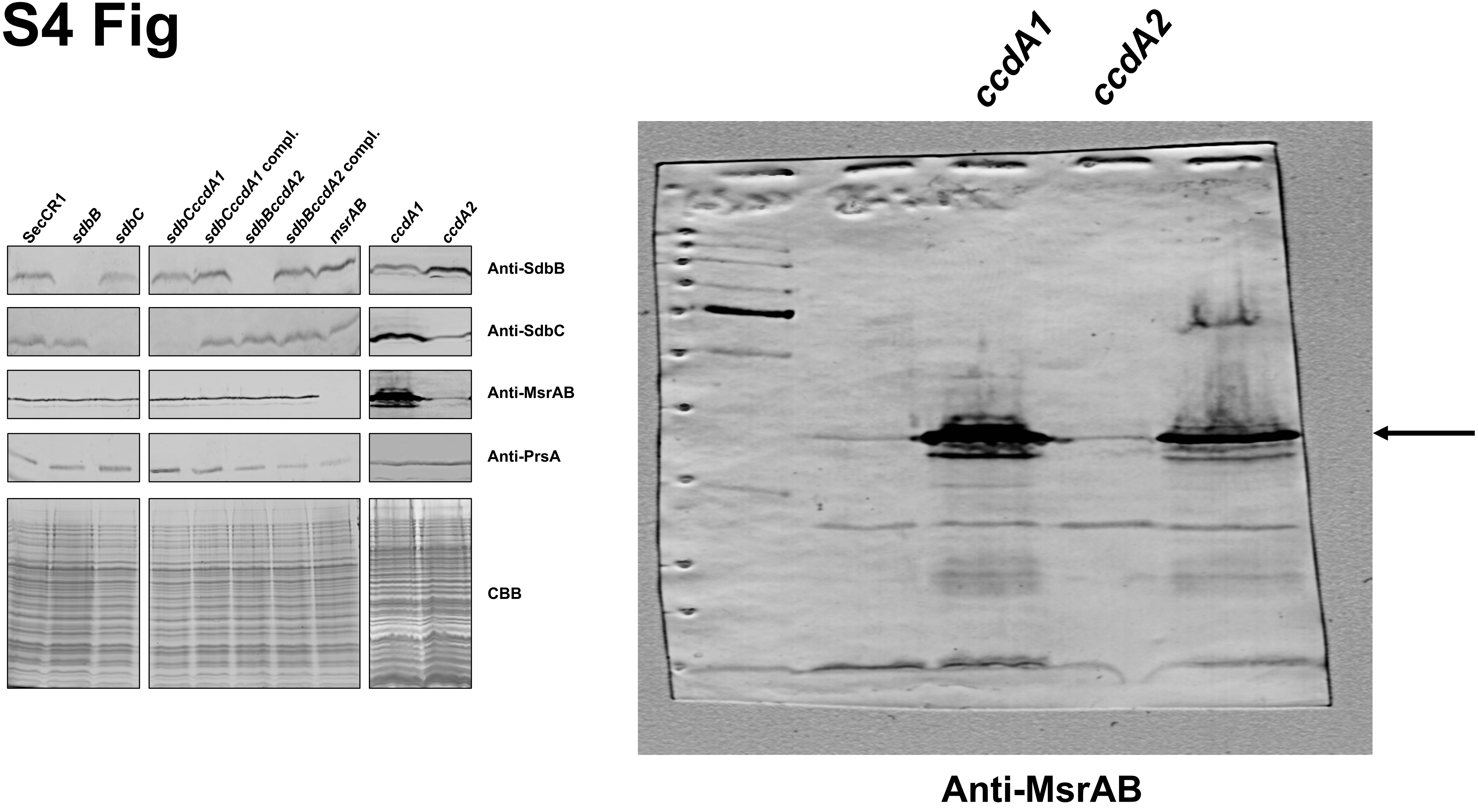
SecCR1
sdbB
sdbC
sdbCccdA1
sdbCccdA1 compl.
sdbBccdA2
sdbBccdA2 compl.
msrAB
ccdA1
ccdA2
Anti-SdbB
Anti-SdbC
Anti-MsrAB
Anti-PrsA
CBB
ccdA1
ccdA2
Anti-MsrAB

S4 Fig

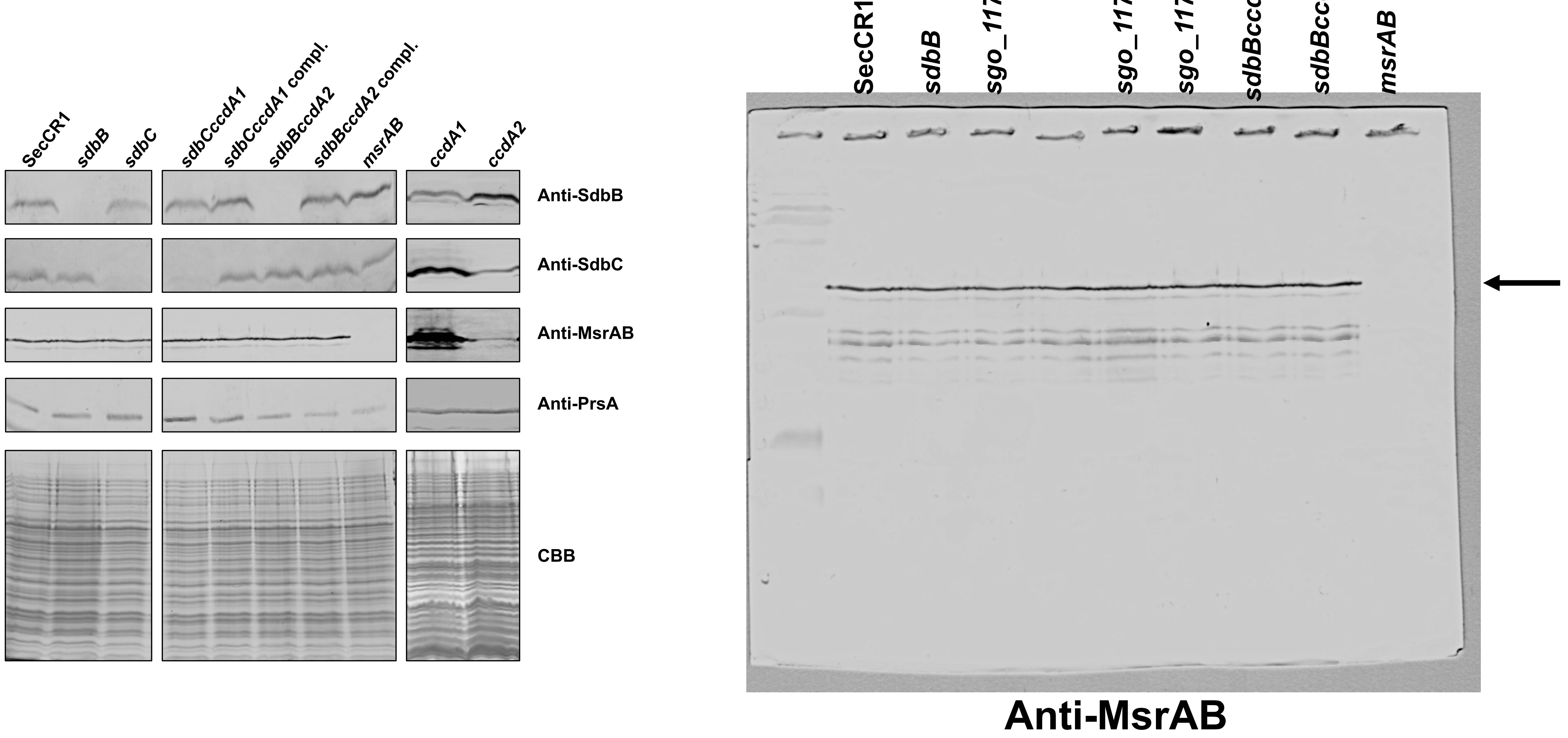

SecCR1
sdbB
sdbC
sdbCccdA1
sdbCccdA1 compl.
sdbBccdA2
sdbBccdA2 compl.
msrAB
ccdA1
ccdA2
Anti-SdbB
Anti-SdbC
Anti-MsrAB
Anti-PrsA
CBB
SecCR1
sdbB
sgo_1177
sgo_1177ccdA1
sgo_1177ccdA1 compl.
sdbBccdA2
sdbBccdA2 compl.
msrAB
Anti-MsrAB

S4 Fig

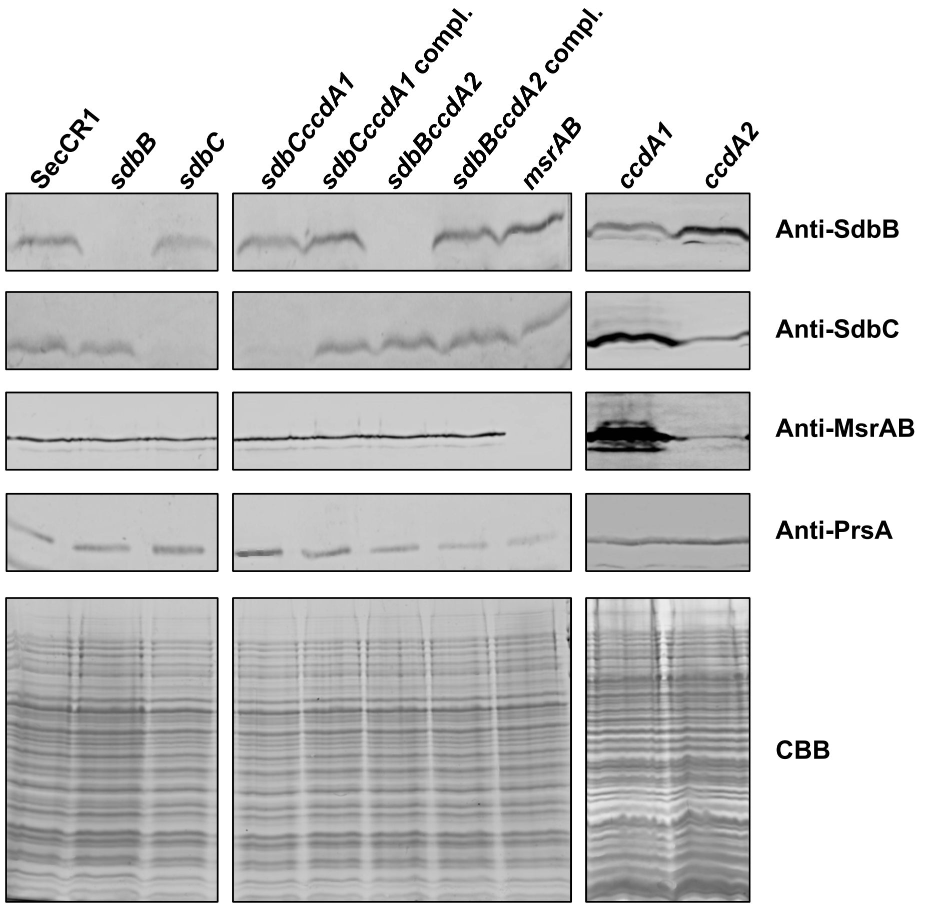

SecCR1
sdbB
sdbC
sdbCccdA1
sdbCccdA1 compl.
sdbBccdA2
sdbBccdA2 compl.
msrAB
ccdA1
ccdA2
Anti-SdbB
Anti-SdbC
Anti-MsrAB
Anti-PrsA
CBB

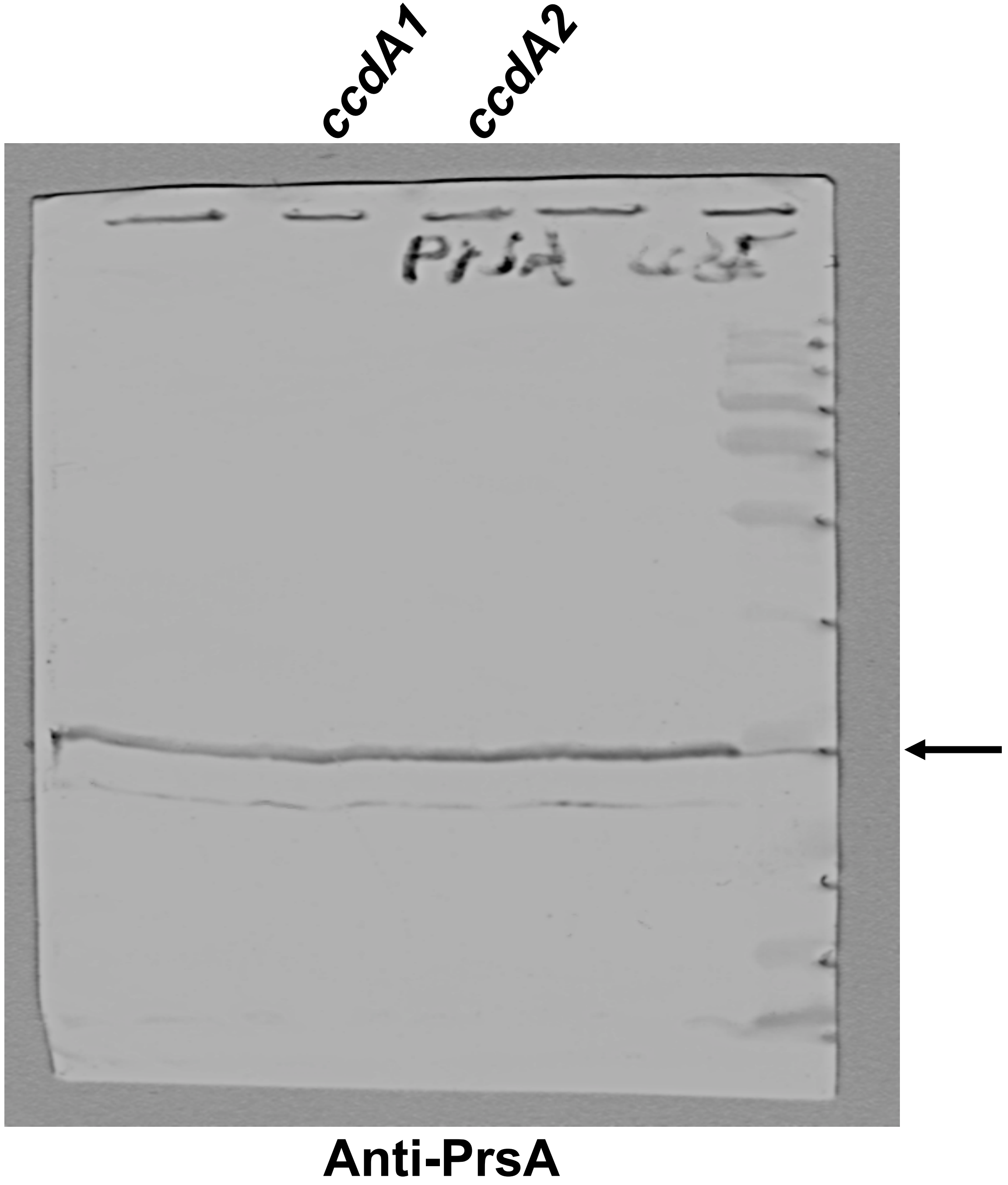

ccdA1
ccdA2
Anti-PrsA

S4 Fig

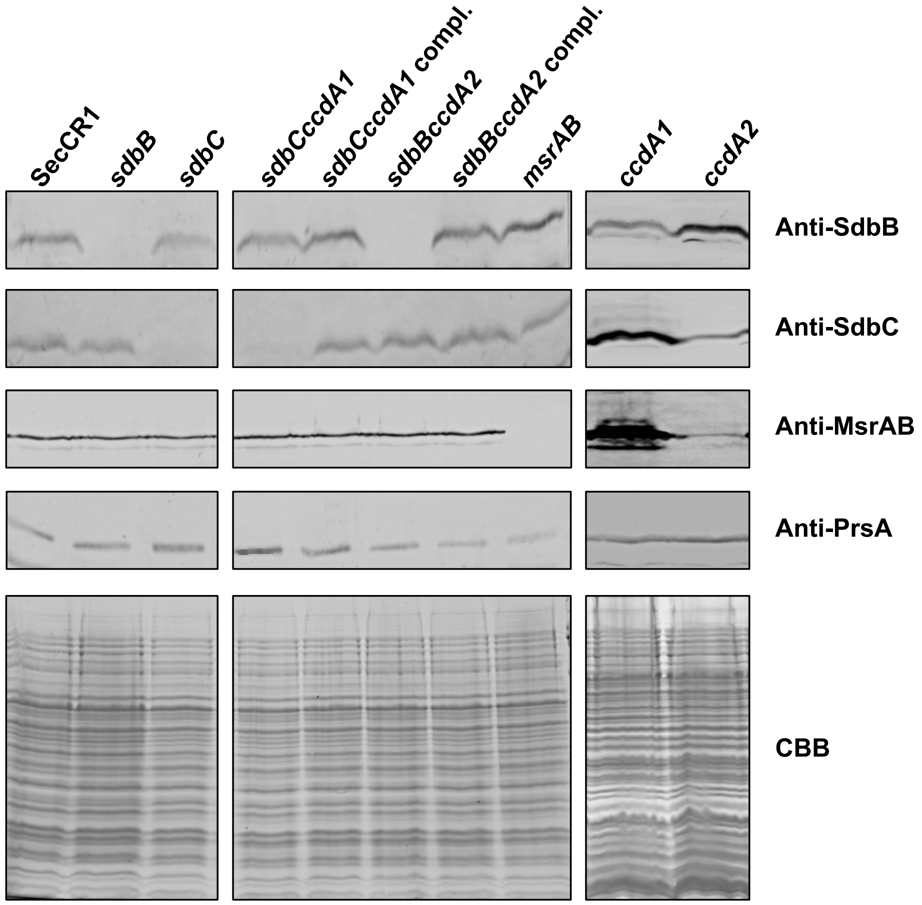
SecCR1
sdbB
sdbC
sdbCccdA1
sdbCccdA1 compl.
sdbBccdA2
sdbBccdA2 compl.
msrAB
ccdA1
ccdA2
Anti-SdbB
Anti-SdbC
Anti-MsrAB
Anti-PrsA
CBB

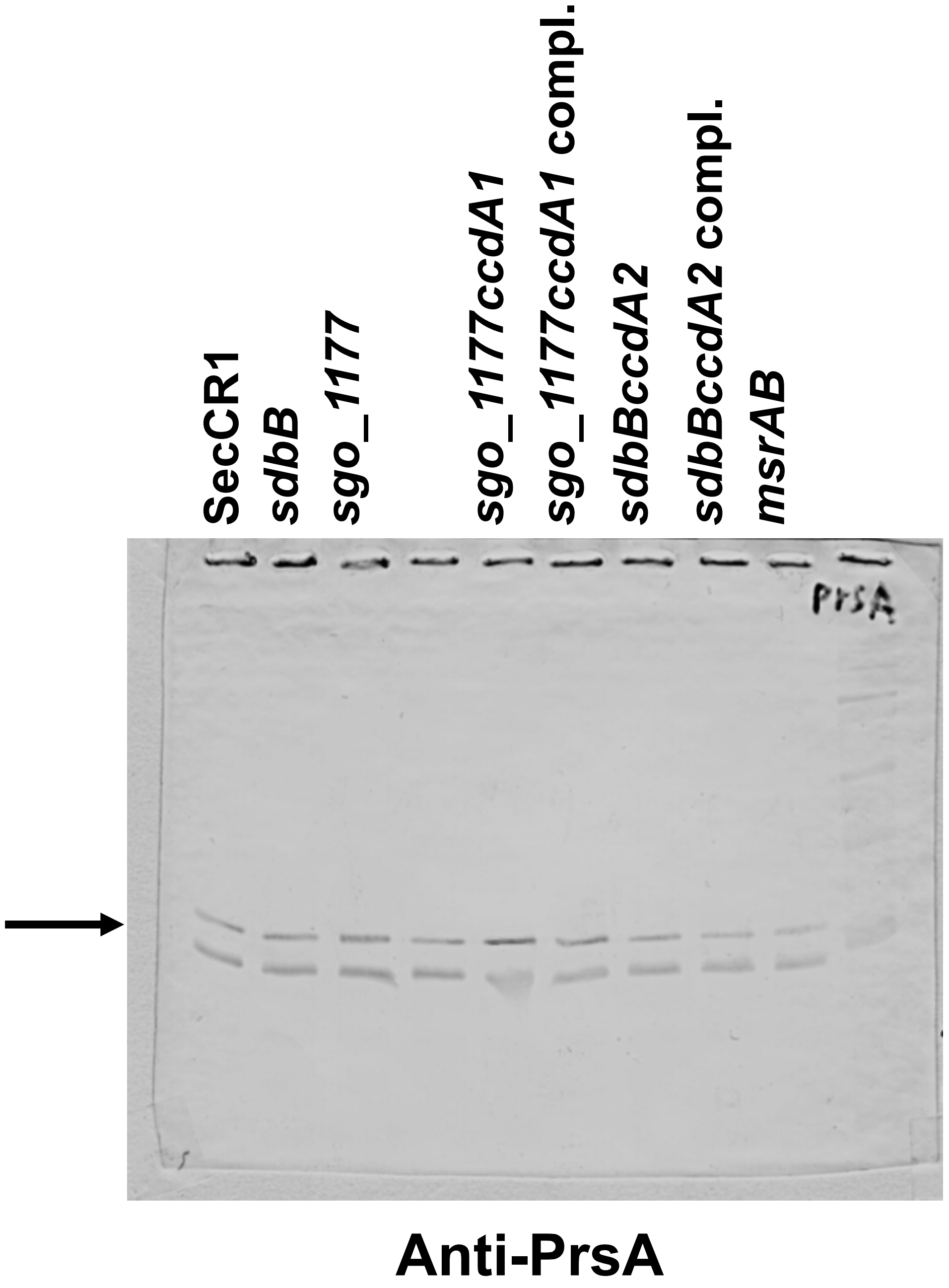
SecCR1
sdbB
sgo_1177
sgo_1177ccdA1
sgo_1177ccdA1 compl.
sdbBccdA2
sdbBccdA2 compl.
msrAB
PrsA
Anti-PrsA

S4 Fig

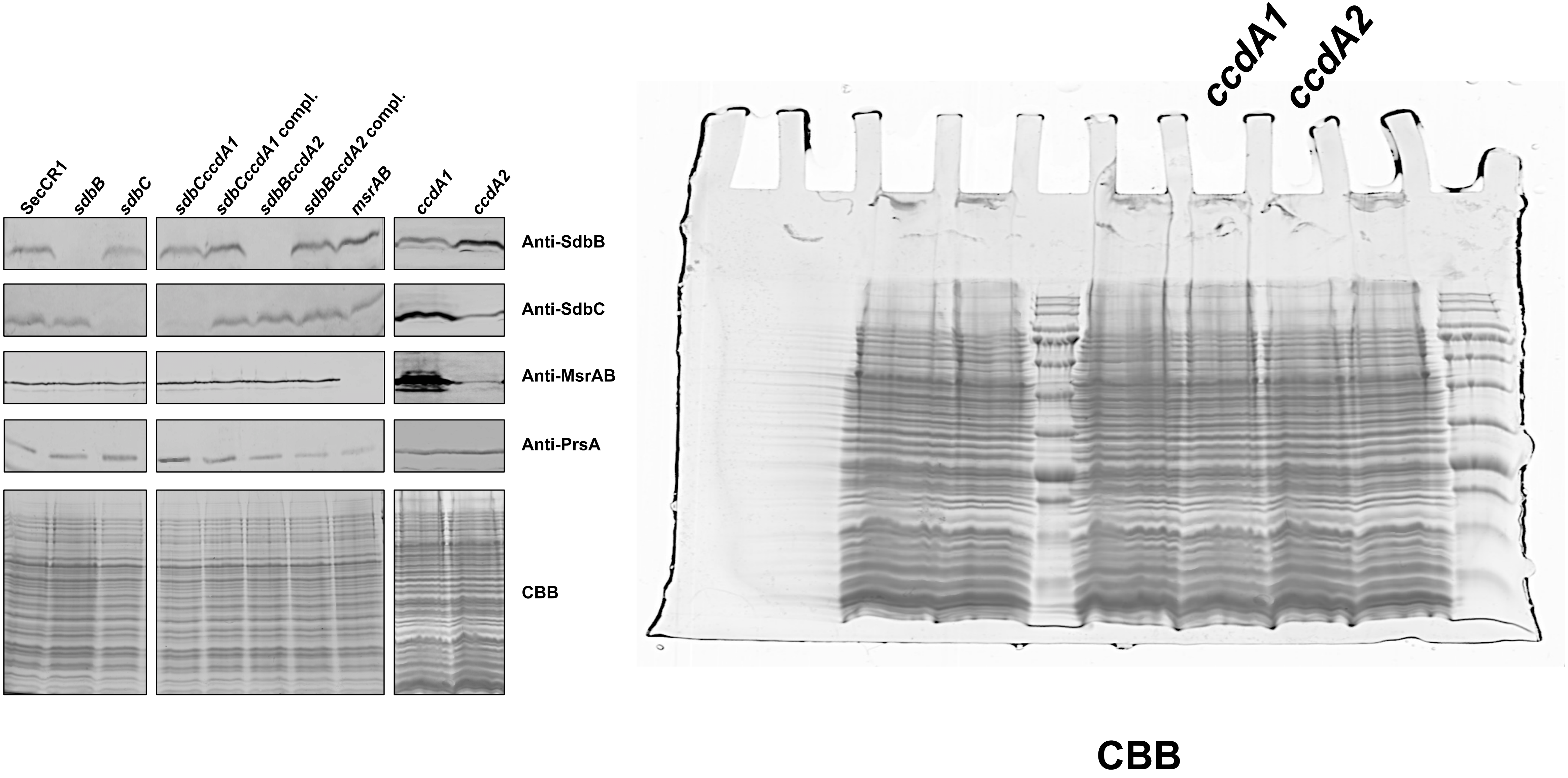
SecCR1
sdbB
sdbC
sdbCccdA1
sdbCccdA1 compl.
sdbBccdA2
sdbBccdA2 compl.
msrAB
ccdA1
ccdA2
Anti-SdbB
Anti-SdbC
Anti-MsrAB
Anti-PrsA
CBB
ccdA1
ccdA2
CBB

S4 Fig

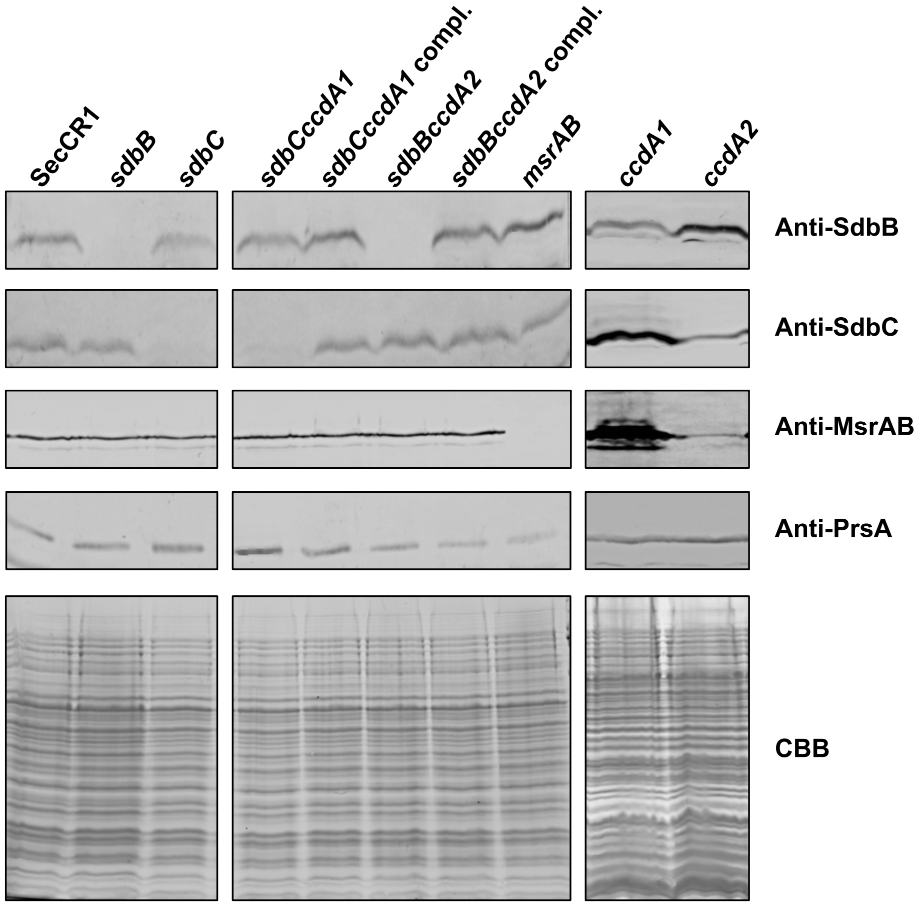
SecCR1
sdbB
sdbC
sdbCccdA1
sdbCccdA1 compl.
sdbBccdA2
sdbBccdA2 compl.
msrAB
ccdA1
ccdA2
Anti-SdbB
Anti-SdbC
Anti-MsrAB
Anti-PrsA
CBB

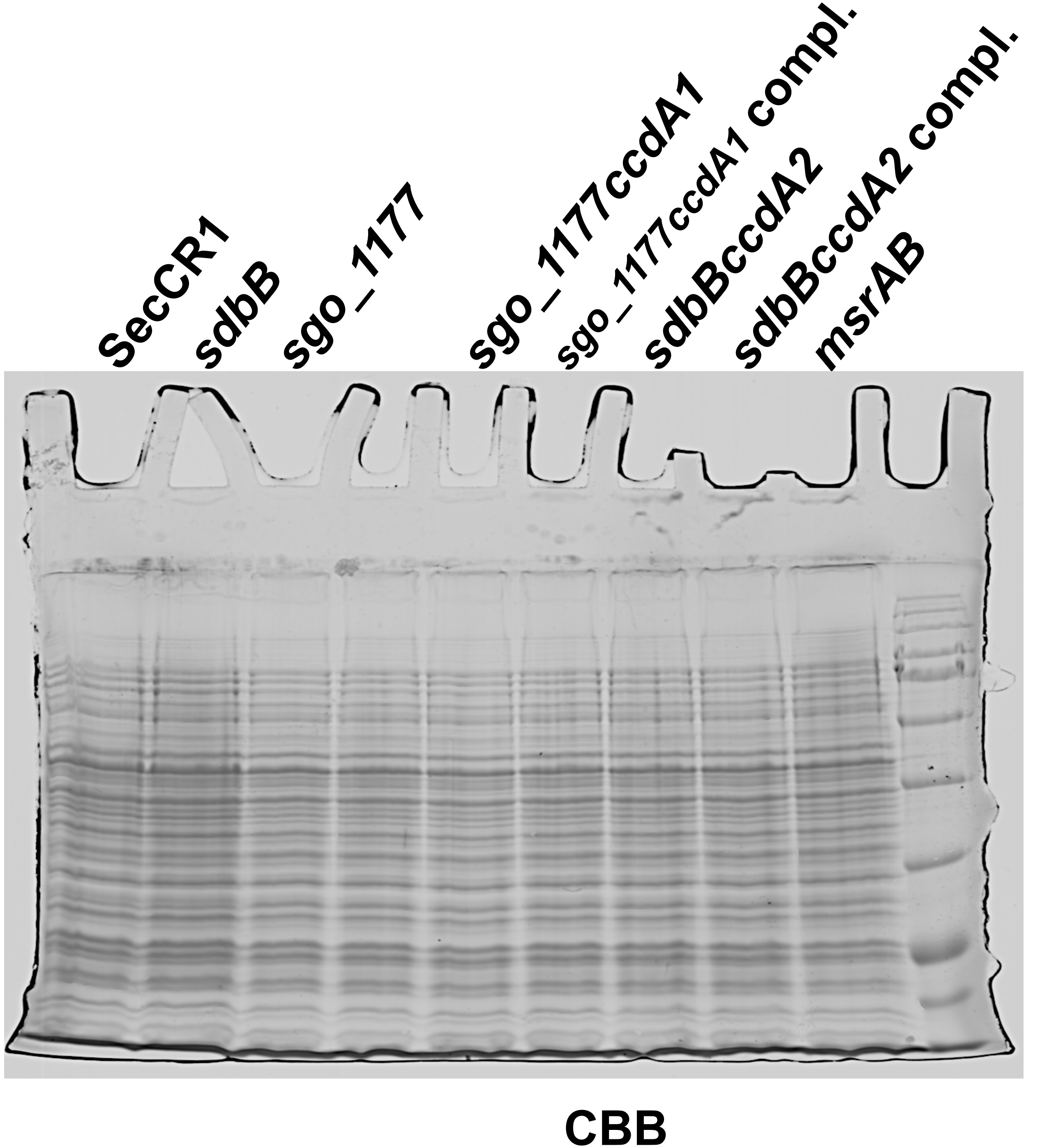
SecCR1
sdbB
sgo_1177
sgo_1177ccdA1
sgo_1177ccdA1 compl.
sdbBccdA2
sdbBccdA2 compl.
msrAB
CBB
