# Supplementary material for: The MsrAB reducing pathway of Streptococcus gordonii is needed for oxidative stress tolerance, biofilm formation, and oral colonization in mice
Source: PLoS One. 2020 Feb 21;15(2):e0229375. doi: 10.1371/journal.pone.0229375 (PMC7034828; doi:10.1371/journal.pone.0229375)
Supplement: S1 Table — (PDF) [file pone.0229375.s006.pdf]

S1 Table. Primers used in this study.

Primer	Gene		Description		Sequence (5'⟶ 3')
SL609	*ermAM*	For	Erythromycin	EcoRV	TGAGATATCCCGGGCCCAAAATTTGTTTGAT
SL729	*ermAM*	Rev	Erythromycin	BamHI	TACGGATCCAGCGACTCATAGAATTATTT
SL801	*aphA3*	For	Kanamycin	BamHI	TACGGATCCGCAAGGAACAGTGAATTGGA
SL823	*aphA3*	Rev	Kanamycin	KpnI	TACGGTACCCAGTTGCGGATGTACTTCAG
SL1146	*msrAB*	For	Knockout		GACACATATTGGCTACATG
SL1147	*msrAB*	For	Knockout	BamHI	TACGGATCCGAAGTACGATAAACCTCTAG
SL1148	*msrAB*	Rev	Knockout		CAGATACTCATCCTCTAC
SL1061	*sgo_1174*	For	Complementation		TGACATATAGTCGGGACAT
SL1235	*sdbB*	Rev	Complementation	SphI	TGAGCATGCTTACTTCATTTCTTTAAAGG
SL666	*pUC19 MCs*	For	Complementation	SphI	ACGCCAAGCTTGCATGCCTGC
SL869	*sdbB*	For	Complementation	BamHI	TACGGATCCGAAAGAAAGACCGCGATTTC
SL1226	*sgo_1170*	Rev	Complementation		GGAACATCACGTGCCCAAG
SL1050	*ccdA1*	For	Complementation		TAAATCTTGGTGTGGGAAAG
SL1307	*ccdA1*	For	Expression	BglII	TACAGATCTGCAACAAGTTTTTTGTTCTTTA
SL1308	*ccdA1*	Rev	Expression	SpeI	TACACTAGTTCCGAATAATGATGCTAAAGC
SL1316	*msrAB*	For	Expression	BamHI	TACGGATCCCGGGTAATCTATCTGGC
SL1319	*msrAB*	Rev	Expression	HindIII	TACAAGCTTTTAAACATAATCCAAAAGATAAC
SL 1464	*msrA*	For	Knockout		TACGGATCCATGGCTGAAATTTATCTAGCAGGC
SL1460	*msrA*	Rev	Knockout	EcoRi	TACGAATTCGTCTTCGGCTAGGACATA
SL1461	*msrA*	For	Knockout	BamHI	TACGGATCCAACAGTTGACGCAAGAGC
SL1462	*msrA*	Rev	Knockout		TACAAGCTTATTTCATGTGTTGAAGTAGA
SL1316	*msrAB-A*	For	Expression	BamHI	TACGGATCCCGGGTAATCTATCTGGC
SL1317	*msrAB-A*	Rev	Expression	HindIII	TACAAGCTTGTAGGCGGCTTGATTAACA
SL1318	*msrAB-B*	For	Expression	BamHI	TACGGATCCAGTGATGAAGAGATTAAGTC
SL1319	*msrAB-B*	Rev	Expression	HindIII	TACAAGCTTTTAAACATAATCCAAAAGATAAC
SL1374	*trxB*	For	Expression	BamHI	TACGGATCCATGTACGATACATTAATTATTG
SL1375	*trxB*	Rev	Expression	HindIII	TACAAGCTTATGAAGGGAAACTTTCAAG
