# Supplementary material for: The MsrAB reducing pathway of Streptococcus gordonii is needed for oxidative stress tolerance, biofilm formation, and oral colonization in mice
Source: PLoS One. 2020 Feb 21;15(2):e0229375. doi: 10.1371/journal.pone.0229375 (PMC7034828; doi:10.1371/journal.pone.0229375)
Supplement: Attachment — Submitted filename: Response to reviews.docx [file pone.0229375.s007.docx]

Point-by-point response to reviews

Reviewer #1: The manuscript describes the MsrAB reducing pathway of Streptococcus gordonii that affects several traits of this species, especially oxidative stress response, biofilm formation in vitro, and mice oral colonization.
The experiments are elegantly designed, the text is well-written, and the figures illustrate the findings adequately. However, there are a couple of minor points that should be clarified, as follow:
- Please clarify why the terminology “oxidative stress resistance” should be used instead of “oxidative stress tolerance”.

Response: Oxidative stress tolerance is a better term. It is now used.

- Line 197. Please revise the Figure citation. It appears that the correct citation would be Fig 5d and 5e, not Fig 5b.

Response: Done. (l. 258)

- Lines 197-199. Please consider adding the graphs for msrA mutant in the figure.

Response: *msrA* was in the figure, i.e. first set of data in Fig 5d and 5e.

- Lines 232-234. The following sentence is a bit awkward and should be revised: "If one reasons that these other proteins are part of a pathway involving MsrAB, then it makes sense that these mutants are also sensitive to these agents."

Response: The sentence has been modified (l. 303-304).

- Line 390: could add the pH and the composition of PBS (e.g., with or without sodium).

Response: pH and composition added (l. 475).

- Line 513: Please include the number of mice tested per experimental group for the competitive oral colonization in mice.

Response: given now (l. 601).

- Lines 531-533. Please revise the statistical analysis description because, for example, only two groups were compared (parental versus mutant strain) for mice oral colonization assay.

Response: The sentence has been revised to state “between the parent and mutant” (l. 620).

Reviewer #2: Authors investigate S. gordonii MsrAB gene, and elucidated its role in oxidative stress tolerance, biofilm formation and colonization in an animal model. A new pathway is proposed involving the MsrAB gene and genes in the adjacent operons. The study is very interesting based on its novelty in the context of S. gordonii. The hypothesis are tested with rigorous experiments and the data is presented clearly.

Response: Thank you for the kind words.

Following are some minor concerns

In the introduction, (line 51) perhaps would be better to give a little more background information about S. gordonii oral colonization and its role in dental plaque formation.

Response: A couple of sentences have been added (l. 49-52).

Indicate whether MsrAB gene is an extension of the MsrA gene sequence. Is MsrAB co-transcribed? Otherwise it is a bit confusing as it says that MsrA is a cytoplasmic protein and MsrAB is an extracytoplasmic protein. MsrA also seem to be sensitive to H2O2 and affects biofilm formation which are similar properties as MsrAB. Therefore it would be better to explain a bit more clearly the differences between MsrA and MsrAB.

Response: MsrAB is a single polypeptide and the gene is located at a separate locus from *msrA*. This has been added to the introduction as well as the gene ID for the two genes (l. 64, l. 71-74). Hopefully, it helps to prevent confusion.

Fig 1a – any explanation for MsrAB to have higher percentage of survival than MsrA.

Response: May be this is because MsrA, as a cytoplasmic enzyme, it plays a role in protecting essential cellular proteins; while MsrAB, as an extracytoplasmic enzyme, it protects mainly surface proteins.

Line 127- indicates data not shown. Please check with journal guidelines regarding policy for data availability.

Response: Data now included as S3 Fig in supplementary file.

Has the biofilm formation been tested under H2O2 and methionine sulfoxide conditions.

Response: No, we did not.

Please indicate the sample size of mice used for the experiment.

Response:. Number of mice per group is now given (l. 601).
